# Force-Triggered Atropisomerization of a Parallel Diarylethene to Its Antiparallel Diastereomers

**DOI:** 10.1021/jacs.3c03994

**Published:** 2023-07-06

**Authors:** Xuancheng Fu, Boyu Zhu, Xiaoran Hu

**Affiliations:** Department of Chemistry, Syracuse University, Syracuse, New York 13244, United States; BioInspired Institute, Syracuse University, Syracuse, New York 13244, United States

## Abstract

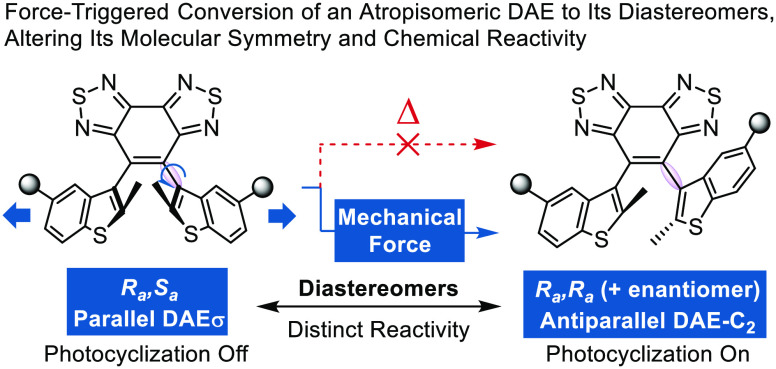

This paper describes a mechanical approach to inducing the atropisomerization of a parallel diarylethene into its antiparallel diastereomers exhibiting distinct chemical reactivity. A congested parallel diarylethene mechanophore in the (*R*_*a*_*,S*_*a*_)-configuration with mirror symmetry is atropisomerized to its antiparallel diastereomers with *C*_2_ symmetry under ultrasound-induced force field. The resulting stereochemistry-converted material gains symmetry-allowed reactivity toward conrotatory photocyclization.

Atropisomers are a subclass of conformers which can be isolated as separate chemical species and which arise from restricted rotation about a single bond.^1^ Atropisomerism is a chiral phenomenon that plays a critical role in various fields of science, including medicinal chemistry,^2^ catalysis,^3^ and materials science.^4^ Diarylethenes (DAEs) are an intriguing class of photoswitchable molecules that exist in the parallel and antiparallel conformer states that usually undergo rapid interconversion.^5^ The antiparallel DAE contains a pair of enantiomers (*R*_*a*_*,R*_*a*_ and *S*_*a*_*,S*_*a*_) with C_2_ rotational symmetry and undergoes reversible conrotatory photocyclization between the ring-open colorless and ring-closed colored states, while its electrocyclization in the ground state is symmetry-forbidden according to Woodward–Hoffmann rules.^6^ In comparison, the parallel *R*_*a*_*,S*_*a*_ conformer is a meso compound with mirror symmetry and is disallowed to cyclize under either thermal or photochemical conditions because of its unfavorable steric interaction and molecular symmetry. Interestingly, Feringa,^7^ Zhu,^8^ and other groups^9^ have found that steric congestion between the DAE ethene bridge and the side arm aryl substituents hinders the rotation around the ethene-aryl single bonds. The two ring-open atropisomers of those crowded DAEs are locked in their respective configurations, and individual atropisomers have been isolated and their properties studied.

The last two decades of research in polymer mechanochemistry have provided a library of stress-responsive molecules known as mechanophores with different functional responses to mechanical stimulation such as color change,^10^ activation of catalysts,^11^ switching of electrical conductivity,^12^ and generation of reactive species.^13^ The fundamental mechanisms behind reported force–matter interactions are summarized into two general categories (Figure 1a): covalent and noncovalent transformations.^14^ Most covalent mechanochemical transformations fall under homolytic cleavage,^15^ heterolytic cleavage,^16^ pericyclic,^17^ or metal-coordinate bond cleavage.^18^ On the other hand, the development of mechanophores harnessing noncovalent transformations is an emerging research topic. For example, Weder and co-workers developed a series of rotaxane- and cyclophane-based supramolecular mechanophores, where force affects the spatial alignment between chromophores and alters their photoluminescent properties.^19^ Saito and co-workers designed “flapping” mechanophores that undergo conformational planarization under mechanical stimulation which extends the π conjugation length.^20^ Similarly, the Matile^21^ and Sommer^22^ groups developed twisted conjugated systems that planarize and gain π-conjugation efficiency under force. Herrmann, Göstl, and co-workers have reported strategies to control supramolecular interactions with force in protein and aptamer materials.^23^ Additionally, several studies have explored mechanochemical methods for inducing atropisomerization between enantiomers.^24^

**Figure 1:**
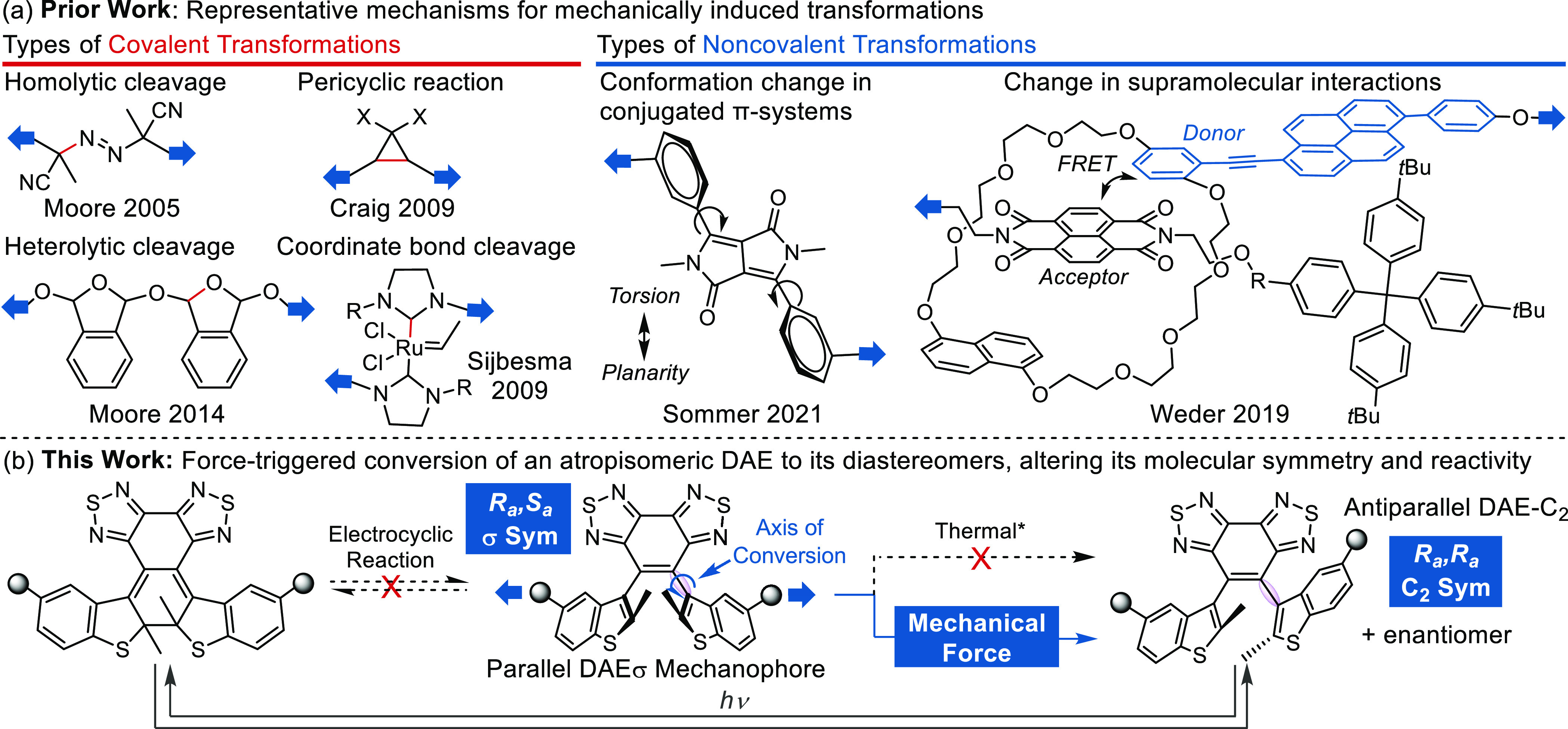
(a) Representative mechanisms for previously reported mechanophores. (b) Mechanical force converts an atropisomeric DAEσ mechanophore to its diastereomers, thereby altering its molecular symmetry and chemical reactivity. *Rotational barrier (thermal): 181 kJ·mol^–1^ (DFT, B3LYP).

Herein we describe a phenomenon of force-stereochemistry coupling that converts an atropisomeric DAE mechanophore to its diastereomers, modifying its molecular symmetry and chemical reactivity (Figure 1b). A sterically congested DAEσ mechanophore with (*R*_*a*_*,S*_*a*_)-configuration and mirror symmetry is locked in the parallel local conformational minimum and is disallowed to undergo a pericyclic reaction. Mechanical force modifies its rotational potential energy surface and triggers the atropisomerization into its antiparallel DAE-C_2_ diastereomers (*R*_*a*_*,R*_*a*_ and *S*_*a*_*,S*_*a*_), rendering C_2_-symmetry-allowed reactivity toward the conrotatory photochemical electrocyclization. The mechanical conversion of an atropisomer to its diastereomeric counterparts represents a general strategy for designing mechanoresponsive materials that are potentially useful in various fields such as stress-sensing and information storage.

We designed the atropisomeric DAE mechanophore based on Zhu and Feringa’s congested DAEs.^7,8^ The *R*_*a*_*,S*_*a*_ parallel DAE is incorporated into a polymer backbone through covalent linkages on the benzothiophenes, enabling the transduction of externally applied mechanical stress to the DAE mechanophore through connected polymers. We chose to connect the polymers at the 5-position of the benzothiophene rings in this preliminary study, although density functional theory (DFT) calculations^25^ using the constrained geometries simulate external force (CoGEF) technique predict that other regioisomers of different pulling positions also exhibit activity toward force-induced atropisomerization (see the SI for details). As demonstrated in Figure 2, elongating the distance between the anchor points results in distortion of the dihedral angle between the benzothiadiazole bridge and the benzothiophene planes, along with bond elongation along the force transduction axis. Eventually, this leads to a sudden rotation of one benzothiophene ring around the ethene-benzothiophene bond, inducing a flip at that chirality axis. As a result, the parent *R*_*a*_*,S*_*a*_ parallel DAEσ is transformed into its antiparallel diastereomer DAE-C_2_ in the *S*_*a*_*,S*_*a*_ (Figure 2) or *R*_*a*_*,R*_*a*_ (not shown) configuration. This atropisomer stereochemistry transformation proceeds with an estimated peak force *F*_max_ of 0.6 nN, which is among the lowest *F*_max_ values calculated by the CoGEF method across reported mechanophores.^25b^ Additionally, the strain energy of the constrained DAE significantly decreases and approaches zero immediately after the conversion (constrained distance = 12.181 Å), owing to the release of a “hidden length” of 7.337 Å.

**Figure 2:**
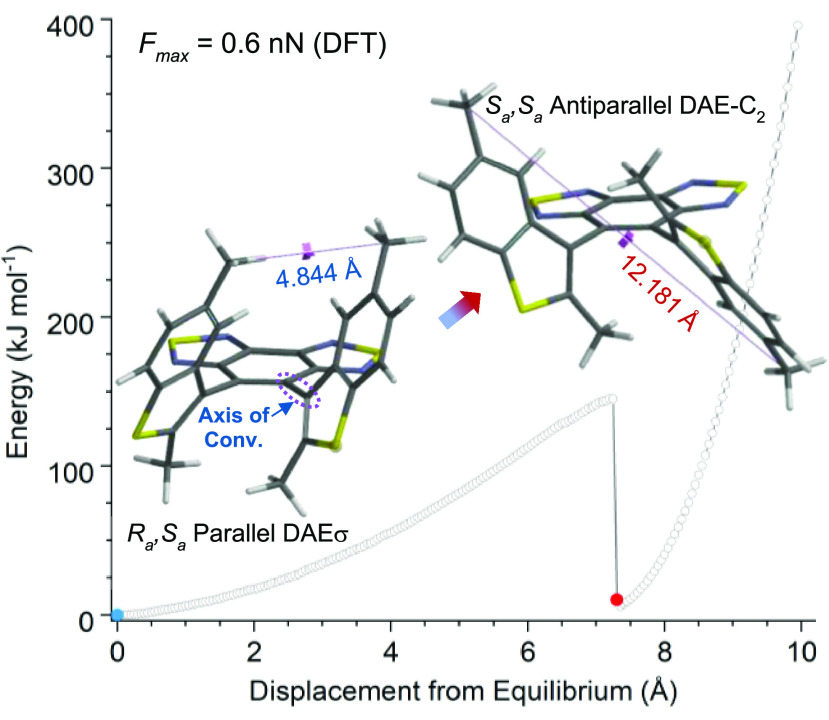
DFT calculation predicts the force-triggered conversion at one of the two stereogenic axes, transforming the *R*_*a*_*,S*_*a*_ DAEσ to its *S*_*a*_*,S*_*a*_ diastereomer DAE-C_2_. Conversion to the *R*_*a*_*,R*_*a*_ stereoisomer (not shown) is equally likely.

With the DFT results supporting our hypothesis of force-triggered atropisomerization into diastereomers, we synthesized the mechanoresponsive materials (Scheme 1) to study the force-stereochemistry coupling experimentally. The synthesis of mechanophore **1σ** started from treating 5-bromo-2-methyl-1-benzothiophene with *n*-BuLi followed by reacting the lithiation product with DMF to yield an aldehyde, which was subsequently reduced with NaBH_4_ to produce alcohol **2**. Alcohol **2** was protected with *tert*-butyldimethylsilyl chloride to yield compound **3** and then brominated with *N*-bromosuccinimide to obtain the organobromine product **4**. **4** was converted to boronic ester **5** by reacting with *n*-BuLi followed by 2-isopropoxy-4,4,5,5-tetramethyl-1,3,2-dioxaborolane. Dibromobenzothiadiazole (BBT) was obtained from the bromination of benzothiadiazole and then reacted with ester **5** through a Suzuki–Miyaura cross-coupling reaction to produce the congested DAE **6** as a mixture of near-equivalent parallel (*R*_*a,*_*S*_*a*_) and antiparallel (*R*_*a*_*,R*_*a*_ and *S*_*a*_*,S*_*a*_) isomers. Atropisomers of **6** do not interconvert and are separated conveniently through silica gel flash chromatography. TBAF deprotection of the desirable parallel **6σ** afforded diol **7σ** (see the Supporting Informatin (SI) for the single crystal structure of **7σ**), which was esterified with α-bromoisobutyryl bromide to produce the target bifunctional initiator **1σ** in the (*R*_*a*_*,S*_*a*_)-configuration. Cu(0)-mediated living radical polymerization of methyl acrylate with initiator **1σ** afforded poly(methyl acrylate) (PMA) **P1σ** (*M*_n_ = 84.4 kDa, *Đ* = 1.19) containing the mechanophore near the center of the polymer chain.

**Scheme 1:**
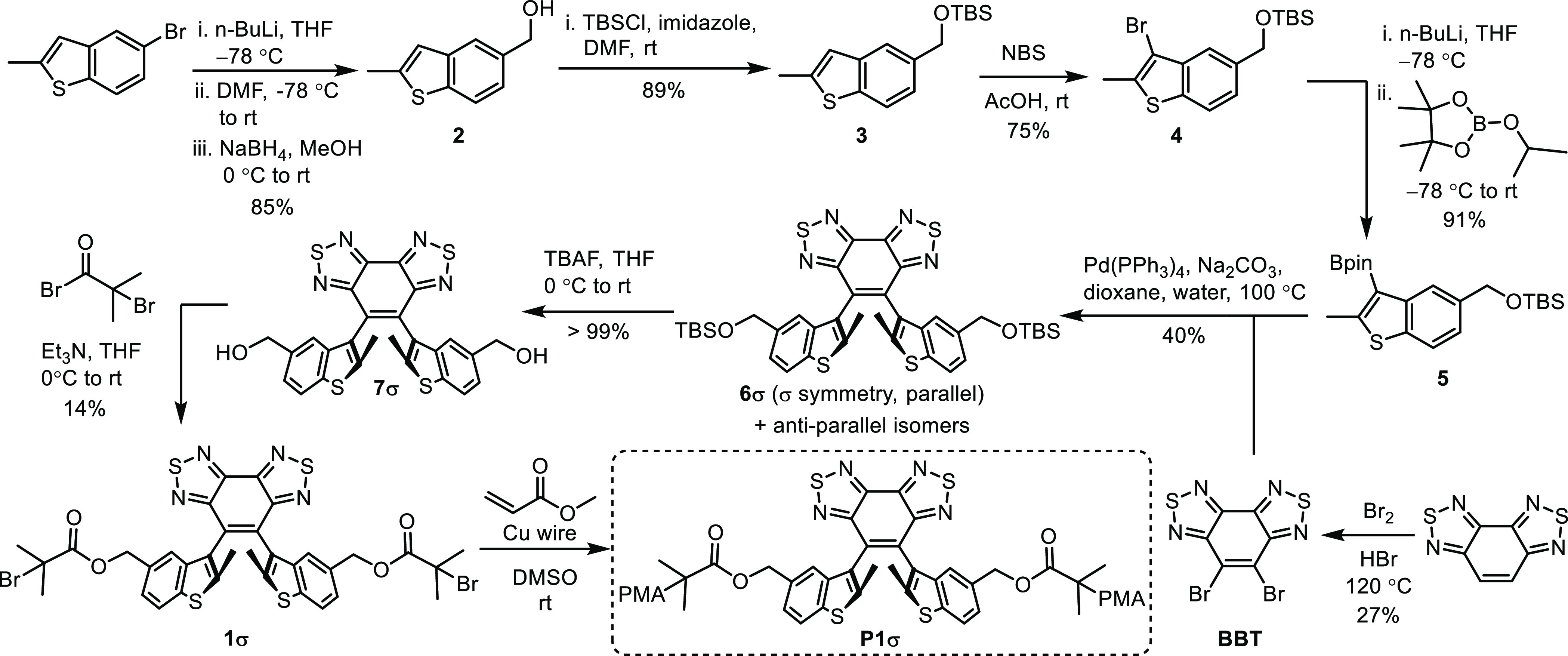
Synthesis of Polymer P1σ Containing a Chain-Centered Parallel DAE Mechanophore with Mirror Symmetry

We studied the mechanical conversion of *R*_*a*_*,S*_*a*_ DAEσ in solution-phase ultrasonication experiments. Ultrasonication is an effective and the most widely adopted technique for studying mechanophore activation in solution.^14^ Ultrasound (U/S) acoustic field causes pressure variations in the solution and generates rapidly collapsing cavitation, inducing a solvodynamic shear force field that transduces force to the backbone of dissolved polymers with the force maximized near the chain center.^22^ An acetonitrile solution of **P1σ** (30 mL, 2.0 mg/mL) was subjected to 1 h pulsed U/S (1 s on/2 s off, 0 °C, 20 kHz), and then the activated polymer was analyzed by NMR spectroscopy. As compared to the ^1^H NMR spectrum of **P1σ**, the ultrasonicated sample shows a new set of resonances at around 7.69 7.08, and 4.96 ppm that match the structure of a separately synthesized control polymer **P1-C**_**2**_ (Figure 3). NMR results provide direct evidence for the conversion of the *R*_*a*_*,S*_*a*_ parallel DAEσ mechanophore to its antiparallel DAE-C_2_ diastereomers upon U/S-induced mechanical activation.

**Figure 3:**
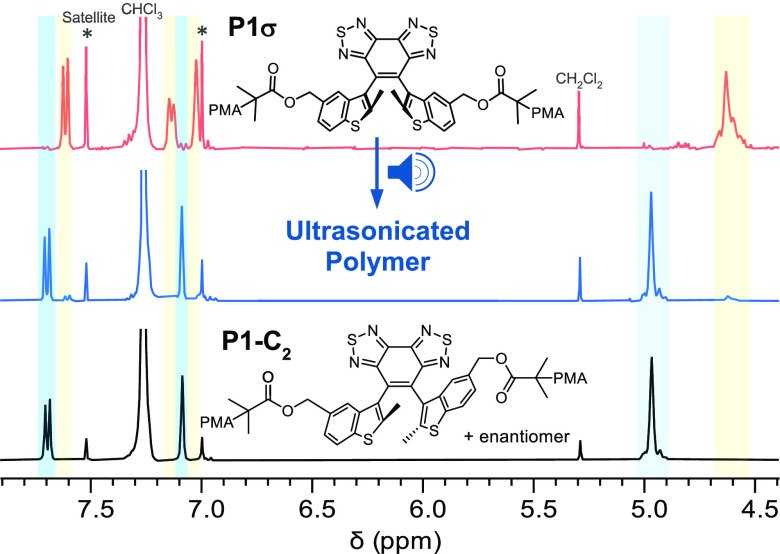
^1^H NMR spectra (400 MHz, CDCl_3_) of **P1σ** before (top) and after (middle) ultrasonication provide direct evidence for the U/S-triggered conversion of the parallel DAEσ to its antiparallel DAE-C_2_ diastereomers. An acetonitrile solution of **P1σ** was subjected to standard ultrasonication conditions, concentrated, and precipitated into cold methanol to afford the ultrasonicated polymer sample for NMR analysis.

As the parallel (*R*_*a*_*,S*_*a*_) and antiparallel (*R*_*a*_*,R*_*a*_ and *S*_*a*_*,S*_*a*_) DAEs are diastereomers and have distinct physiochemical properties,^5^ we studied the excited-state reactivity of **P1σ** as a function of ultrasonication time using UV–vis spectroscopy (Figure 4). A **P1σ** solution (12 mL, 2.0 mg/mL in acetonitrile) was subjected to standard ultrasonication conditions. After each duration of ultrasonication, aliquots of the solution were removed and analyzed. The initial polymer **P1σ** containing the *R*_*a*_*,S*_*a*_ DAEσ mechanophore was colorless and photoinert. The polymer solutions remained colorless after sonication as the conversion of stereochemistry was not expected to affect its visible absorption. Notably, UV irradiation (λ = 365 nm) turned the ultrasonicated polymer sample into a red color, with an absorption peak emerging at around 520 nm. The photostationary state (PSS) (DAE_closed_% = 85%) was achieved after about 40 s irradiation using a hand-held UV lamp (see the SI for details). Moreover, the ultrasonicated solution could be switched between the colored and colorless forms reversibly under UV and visible irradiation, respectively. We observed minimal fatigue after five cycles of UV irradiation at 365 nm, followed by three cycles at 254 nm. The photochromic property and the absorption profile of the U/S-activated samples match the antiparallel model materials **(±)-7C**_**2**_ and **P1-C**_**2**_ (see the SI for details), supporting the hypothesis that U/S activation converts the photoinert *R*_*a*_*,S*_*a*_ parallel DAEσ into its photoswitchable antiparallel DAE-C_2_ diastereomers (*R*_*a*_*,R*_*a*_ and *S*_*a*_*,S*_*a*_). Additionally, the photochromic property of the U/S-activated materials is persistent over the course of this project, indicating the antiparallel DAE-C_2_ product does not thermally atropisomerize to the parent parallel form. The permanent change of the atropisomer stereochemistry, molecular symmetry, and chemical reactivity of our DAEσ mechanophore is distinct from other noncovalent mechanophores that undergo transient conformational changes under force.

**Figure 4:**
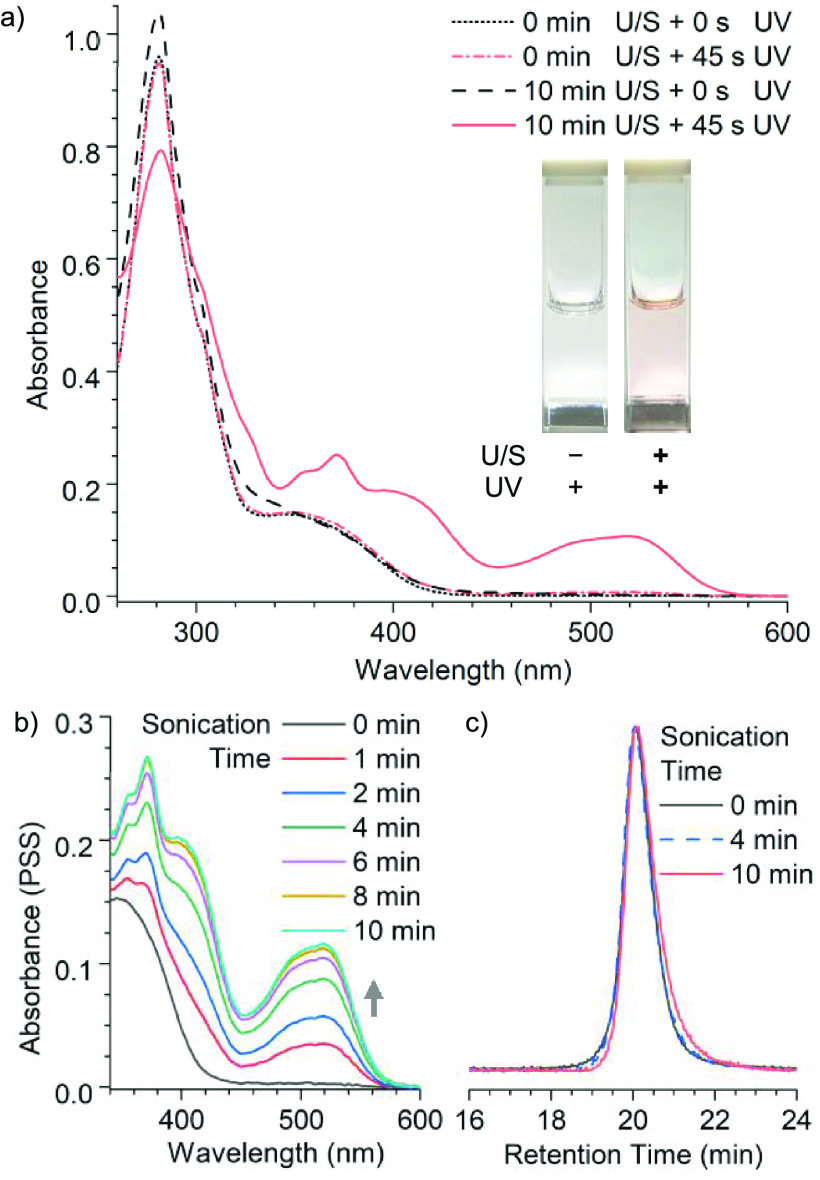
(a) Ultrasonication-dependent photochromism of **P1σ** demonstrates that force triggers the conversion of the photoinert parallel *R*_*a*_*,S*_*a*_ DAEσ mechanophore into its photochromic antiparallel DAE-C_2_ diastereomers. The resulting material gains symmetry-selective reactivity to photoswitch between the ring-open colorless (black dashed) and ring-closed colored forms (red solid). (b) Absorption of P**1σ** solutions in PSS as a function of ultrasonication time indicates the conversion plateaued after ∼10 min ultrasonication. PSS was achieved by 45 s irradiation (365 nm) under a hand-held UV lamp. (c) Normalized GPC traces of **P1σ** as a function of ultrasonication.

The molecular weights of the ultrasonicated polymers were monitored by gel permeation chromatography (GPC). The force-triggered atropisomerization of the DAE mechanophore is a noncovalent process and requires an unusually low magnitude of force according to DFT (*vide supra*) and experimental results (Figures S9 and S10). Indeed, the conversion rapidly approached a plateau after ∼10 min ultrasonication as demonstrated by the photostationary-state absorption spectra of samples as a function of ultrasonication time (Figure 4), whereas we only observed a slow decrease in the polymer molecular weight and slight broadening of the polydispersity over the same period (as opposed to scissile mechanophores that result in bimodal distributions upon activation).^15a^

To confirm the observed transformation is mechanically triggered, we synthesized a control polymer **P2** containing the *R*_*a*_*,S*_*a*_ DAEσ moiety at the polymer chain-ends and subjected it to identical ultrasonication conditions. (See the SI for details.) The control polymer **P2** remained photoinert before and after ultrasonication. Since the chain-end DAEσ moiety in **P2** experienced the same ultrasonication conditions but little force is transduced to the polymer chain-ends, this control experiment confirms that the observed structure and property changes in our system are indeed induced by the applied force.

In summary, this paper introduces a new strategy for converting an atropisomeric mechanophore to its diastereomers, thereby regulating its chemical reactivity. As a proof-of-concept demonstration, a congested *R*_*a*_*,S*_*a*_ parallel DAE molecule is converted to its antiparallel diastereomers in the *R*_*a*_*,R*_*a*_ and *S*_*a*_*,S*_*a*_ configurations under U/S-induced force field. Concomitantly, the converted material is bestowed with *C*_*2*_ symmetry and acquires symmetry-allowed reactivity to undergo a conrotatory photocyclization. This mechanical approach to regulating atropisomer stereochemistry, molecular symmetry, and chemical reactivity contributes new insights into the fundamentally important atropisomerization phenomenon and could lead to the development of new mechanoresponsive materials that are potentially useful in applications including stress-sensing and information storage.

## Supporting Information Available

Experimental details, synthetic procedures, DFT calculations, GPC data, UV–vis and NMR spectra (PDF)
