# Supplementary material for: Force-Triggered Atropisomerization of a Parallel Diarylethene to Its Antiparallel Diastereomers
Source: J Am Chem Soc. 2023 Jul 6;145(29):15668–73. doi: 10.1021/jacs.3c03994 (PMC10375474; doi:10.1021/jacs.3c03994)
Supplement: Supplementary file 1 — ja3c03994_si_001.pdf [file ja3c03994_si_001.pdf]

Supporting Information

Force-Triggered Atropisomerization of a Parallel Diarylethene to Its Antiparallel Diastereomers

Xuancheng Fu, Boyu Zhu, Xiaoran Hu*

Department of Chemistry and BioInspired Institute, Syracuse University, Syracuse, New York 13244, United States.

*To whom all correspondence should be addressed. E-mail: xhu156@syr.edu

Contents

1. General Considerations 2
2. DFT Calculations 3
3. Determination of Photostationary State 4
4. Supplementary Figures 5
5. Synthetic Details 11
6. NMR Spectra 18
7. Single Crystal X-ray Diffraction 32

1. General Considerations

All reactions were conducted under standard air-free conditions under an atmosphere of nitrogen gas with magnetic stirring unless otherwise mentioned. All reactants and solvents were purchased from commercial suppliers and used without further purification unless otherwise noted. Flash chromatography was performed on a Biotage Isolera System with Yamazen Corp. universal silica gel columns (Pore Size 60 angstroms, Particle Size 40-63 microns).

NMR spectra were acquired on a Bruker Avance III HD 400 MHz spectrometer. ^{1}H NMR spectra are reported relative to residual protonated solvent (7.26 ppm for $CHCl_3$). ^{13}C NMR spectra are reported relative to solvent signals (77.16 ppm for $CHCl_3$). Multiplicity abbreviations are as follows: s = singlet, d = doublet, t = triplet, q = quartet, dd = doublet of doublets, ABq = AB quartet, m = multiplet, br = broad.

Mass spectra were acquired on a DART-SVP (Direct Analysis in Real Time) ion source (IonSense, Saugus, MA) coupled to an Exactive Orbitrap mass spectrometer (Thermo Scientific, Bremen, Germany) at the Cornell Chemistry Mass Spectrometry Facility.

Molecular weight distributions of polymers were measured at the Michael Szwarc Polymer Research Institute at SUNY College of Environmental Science and Forestry, using Waters size-exclusion chromatography line (SEC) in THF at a flow rate of 0.8 mL/min. Fourteen poly(styrene) standards (Polymer Standards Service) were used for calibration.

All solution optical spectra were acquired of samples in quartz cuvettes. Electronic absorbance spectra were acquired with an Evolution 201 UV-visible spectrophotometer in double-beam mode using a solvent-containing cuvette for background subtraction spectra of solution samples. Fluorescence spectra were measured with an Agilent Cary Eclipse G9800A Fluorescence Spectrophotometer (Figure S8) or a Horiba Scientific FluoroMax Spectrofluorometer (Figure S9).

Ultrasound experiments were performed using a Vibra Cell 505 liquid processor equipped with a 13 mm full wave solid probe (254 mm long, Sonics, part #630-0217), sonochemical adapter (Sonics, part #830-00014), and a 10-50 mL reaction vessel (Sonics, part #830-00012). All sample solutions were purged with argon for 20 minutes prior to ultrasonication. Argon gas bubbling is continued through ultrasonication experiments. The reaction vessel was immersed in an ice bath during the ultrasonication. The ultrasound treatments were performed in pulse mode (1s on/2s off) with 20% amplitude. All reported sonication times are "sonication-on" time.

UV irradiations at 365 nm or 254 nm were conducted using a hand-held UV lamp (Chemglass, part # CLS-1625). The lamp wattage is 6 watts. Visible light irradiations were performed using an iPhone flashlight.

2. DFT Calculations

Density Functional Theory (DFT) calculations using the constrained geometries simulate external force (CoGEF) technique were performed on Spartan '20 at B3LYP/6-31G* level of theory (*J. Chem. Phys.* **2000**, 112, 7307-7312; *J. Am. Chem. Soc.* **2020**, 142, 16364-16381). A truncated DAEs structure was first equilibrated. Starting from this local energy minimum (relative energy = 0 kJ/mol), the distance between the two terminal methyl groups was increased in small incremental steps (0.05 Å per step), and the energy of the molecule was minimized at each step. The force at each elongation step was calculated from the slope of the energy-strain curve. Figure S1 shows the DFT calculation for the 5-substituted DAE which is the focus of this study. Although DFT results predict that the 4-, 6-, and 7-substituted regioisomers are also active, we will report DFT and experimental results in a separate study. The unusually low maximum force suggests the atropisomeric DAE mechanophore is highly mechanosensitive. The mechanosensitivity of this type of stereochemistry-converting mechanophores is supported by preliminary experimental results (Figure S9 and Figure S10) and will be systematically investigated in follow-up studies.

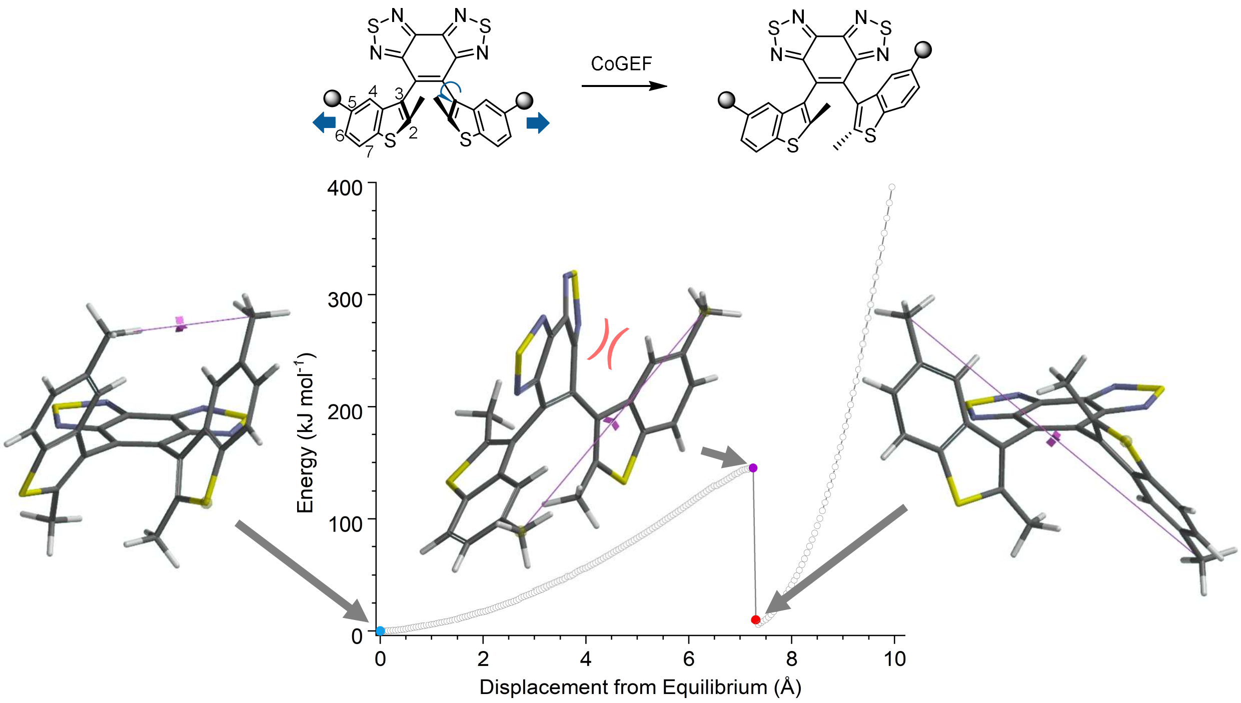

Figure S1a. The structure of the truncated 5-substituted DAE at equilibrium geometry (left), immediately prior to the stereochemistry conversion (middle), and immediately after the mechanical conversion (right). The maximum force 0.6 nN was calculated from the slope of the curve.

The rotational barrier under thermal conditions was computed using DFT calculations at the B3LYP/6-31G* level of theory. The dihedral angle between the ethene bridge and a

benzothiophene was increased in small incremental steps (1° per step), and the energy of the molecule was minimized at each step.

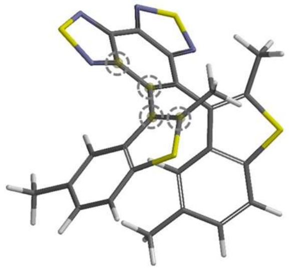

3. Determination of Photostationary State

The ring-closed DAE **(±)-1C$_2^{closed}$** was synthesized, isolated, and structurally characterized (see the Synthetic Details). A dilute solution of the ring-closed DAE in acetonitrile was prepared in the dark, and its optical density value OD_{closed} at 519 nm was measured by UV-vis spectroscopy to be 0.1024. This colored solution was irradiated with a white flashlight for 5 min, triggering the ring-opening reaction and the complete discoloration of the solution. The resultant solution was then irradiated under a hand-held UV lamp at 365 nm for 2 min to achieve its photostationary state (PSS). The UV-vis absorption spectrum of the PSS solution was measured, and its optical density value OD_{PSS} at 519 nm was determined to be 0.0874. The percentage conversion in the PSS was calculated: $DAE_{closed}\% = OD_{PSS}/OD_{closed} = 85\%$.

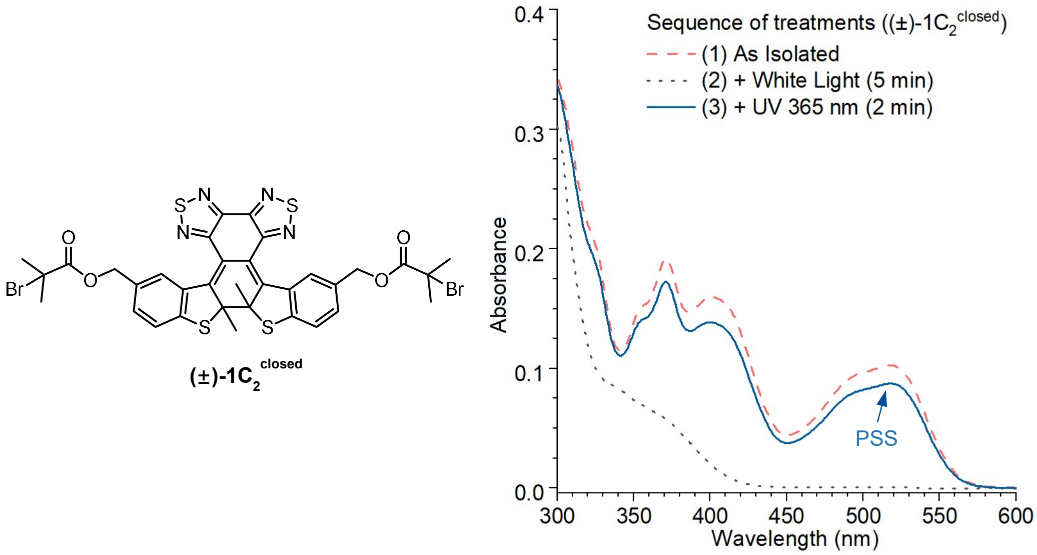

Figure S2. UV-vis absorption spectra of a dilute acetonitrile solution of **(±)-1C$_2^{closed}$** after a sequence of manipulations – Red dashed line: as isolated **(±)-1C$_2^{closed}$** dissolved in acetonitrile; Black dotted line: after 5 min visible light irradiation; Blue solid line (PSS): after 2 min UV irradiation at 365 nm. The percentage conversion in the PSS was calculated: $DAE_{closed}\% = OD_{PSS}/OD_{closed} = 85\%$.

4. Supplementary Figures

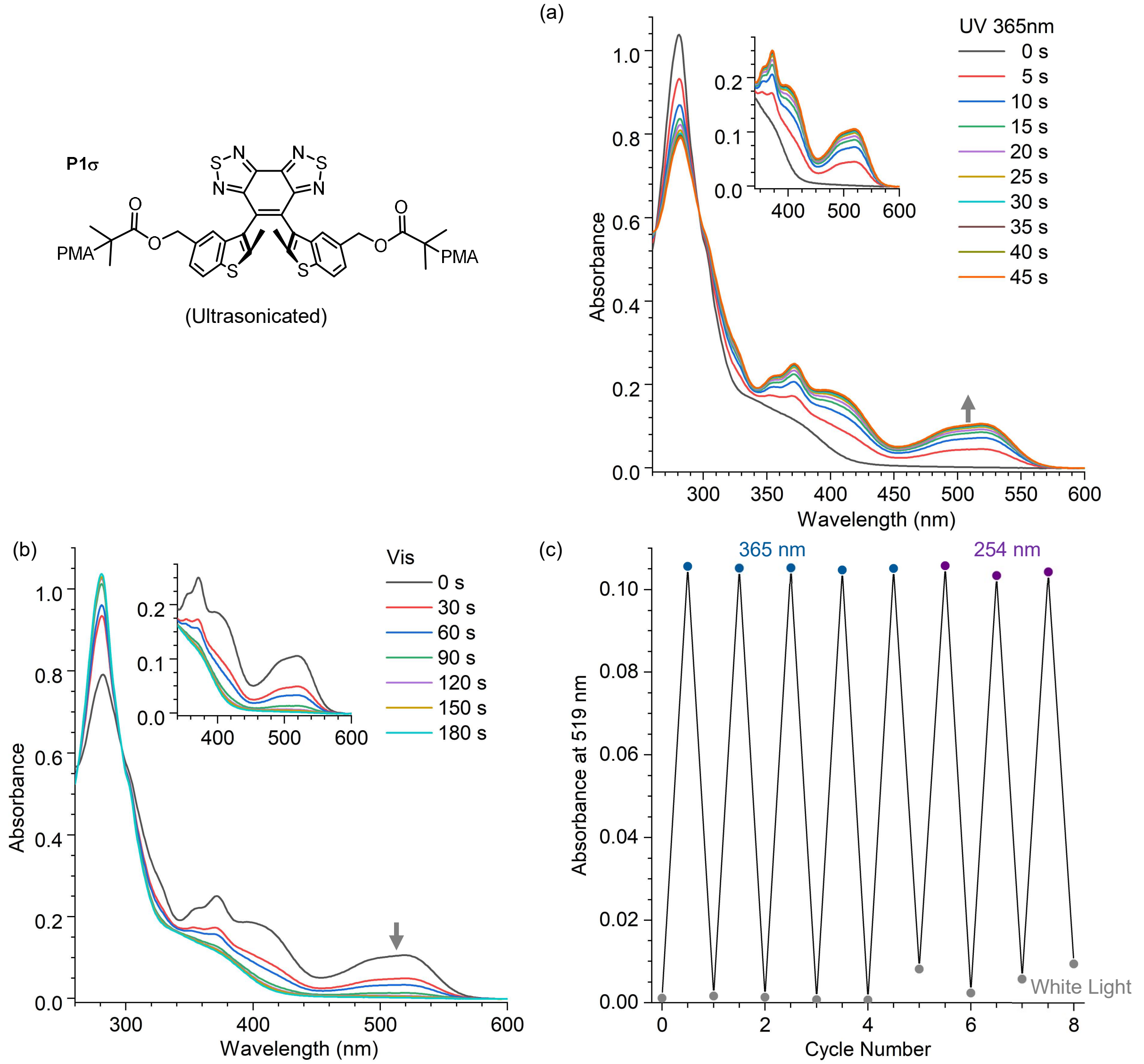

Figure S3. The chemical structure of polymer **P1σ** and its photochemical property after 10 min ultrasonication (2 mg/mL in acetonitrile) characterized by UV-vis spectroscopy. (a) Photochromism of the **P1σ** solution under 365 nm UV irradiation. The PSS was achieved after about 40 s irradiation. (b) Subsequent visible-light irradiation of this polymer solution resulted in the ring-opening reaction and discoloration. The color completely disappeared after about 150 s visible irradiation using a white flashlight. (c) Reversible photochromism of the sonicated **P1σ** solution under UV (λ = 365 nm or 254 nm) and visible light. The absorbance was monitored at the absorption peak of the ring-closed DAE at 519 nm.

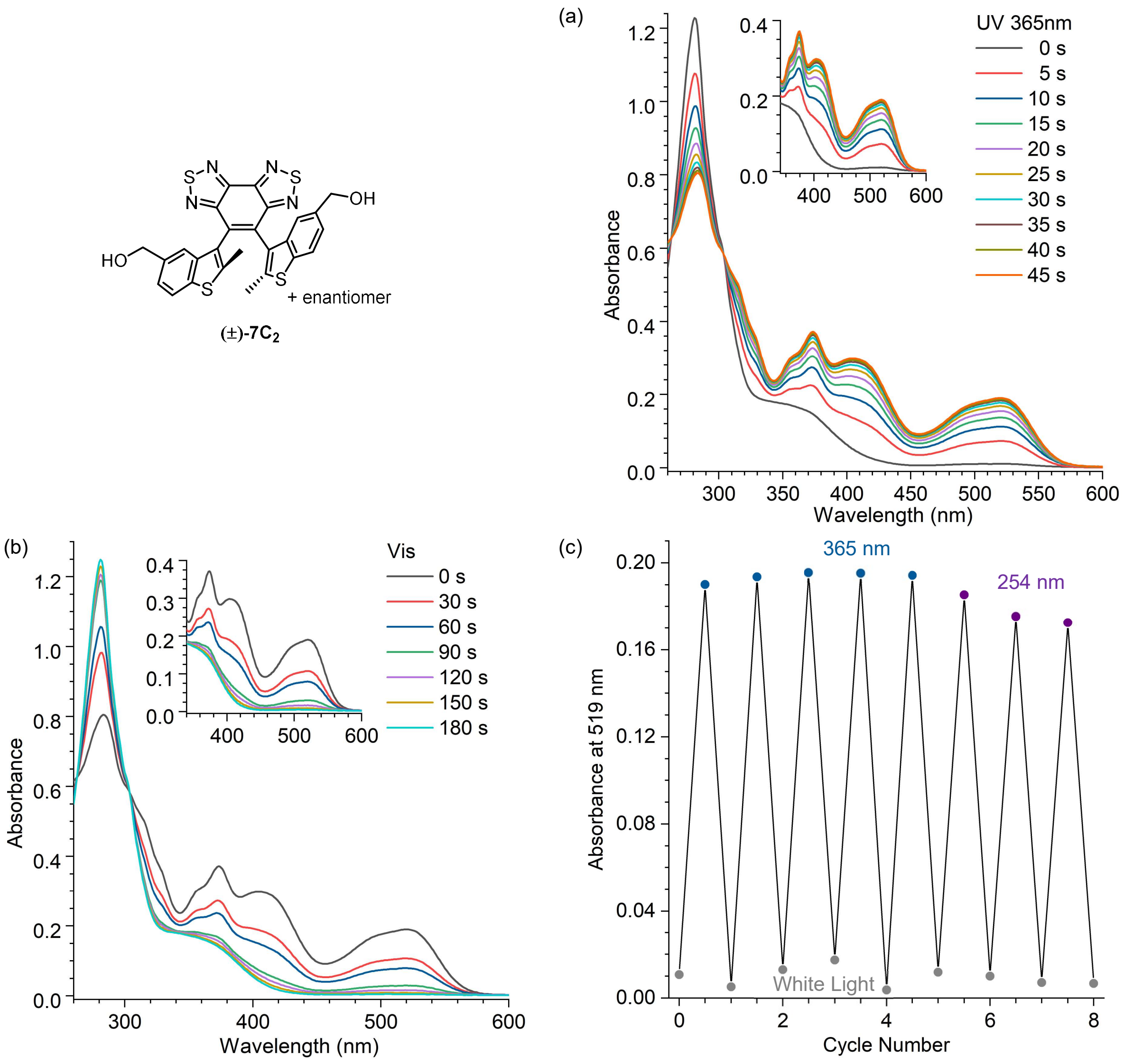

Figure S4. The chemical structure of a model photoswitch molecule **(±)-7C$_2$** and its photochemical property in acetonitrile characterized by UV-vis spectroscopy. (a) Photochromism of the **(±)-7C$_2$** solution under 365 nm UV irradiation. The PSS was achieved after about 40 s irradiation. (b) Subsequent visible-light irradiation of this **(±)-7C$_2$** solution resulted in the ring-opening reaction and discoloration. The color disappeared completely after about 150 s visible irradiation under a flashlight. (c) Reversible photochromism of **(±)-7C$_2$** under UV (λ = 365 nm or 254 nm) and visible light. The absorbance was monitored at the absorption peak of the ring-closed DAE at 519 nm.

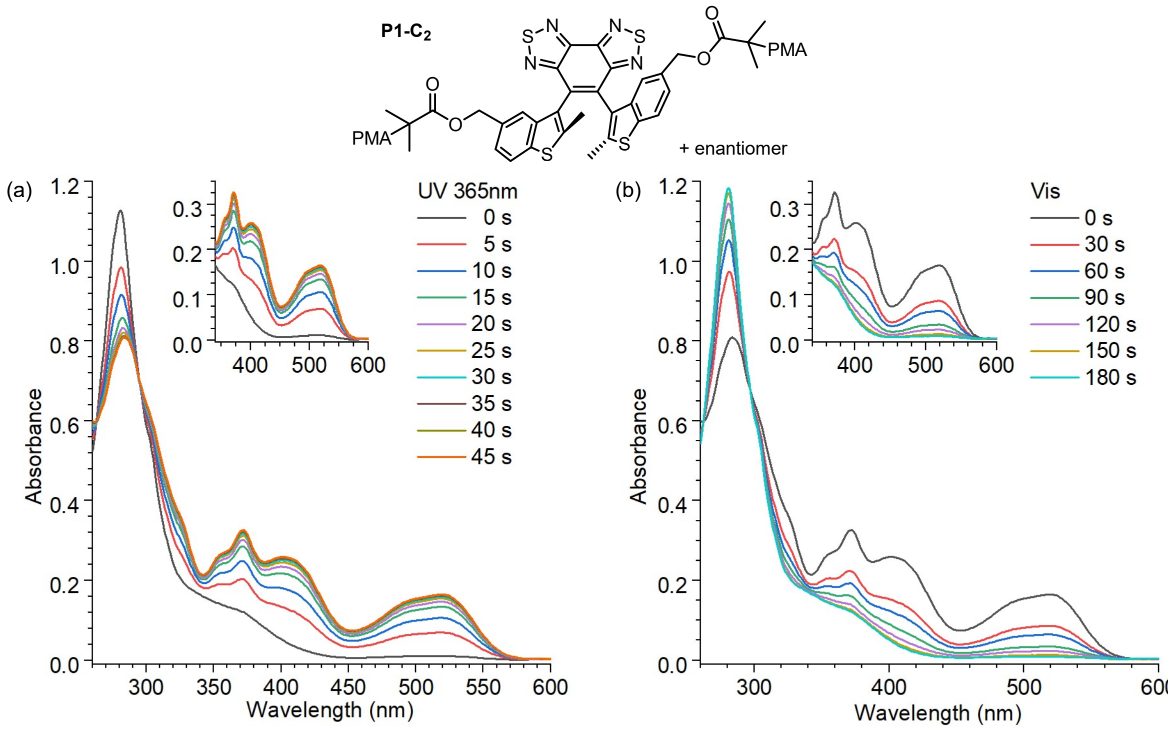

Figure S5. The chemical structure of polymer **P1-C₂** and its photochemical property in acetonitrile characterized by UV-vis spectroscopy. (a) Photochromism of the **P1-C₂** solution under 365 nm UV irradiation. The PSS was achieved after about 40 s of irradiation. (b) Subsequent visible-light irradiation of this polymer solution resulted in the ring-opening reaction and discoloration. The color disappeared completely after about 150 s of visible irradiation.

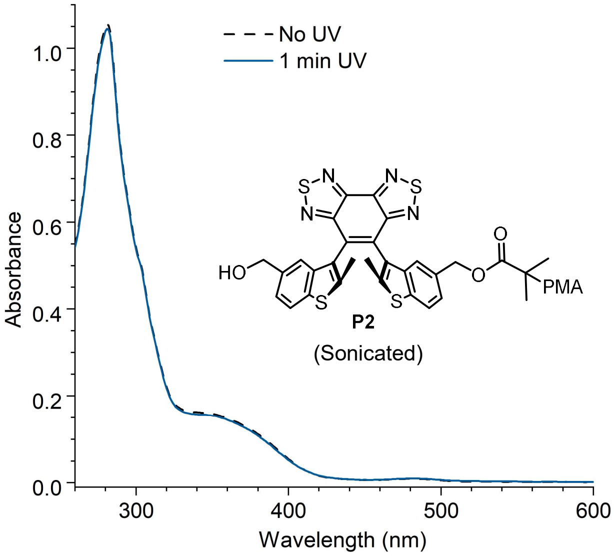

Figure S6. UV-vis absorption spectra of an ultrasonicated acetonitrile solution of control polymer **P2** (2 mg/mL, 10 min ultrasonication) before and after UV irradiation (λ = 365 nm, 1 min). The chain-end control polymer remained photoinert after 10 min ultrasonication.

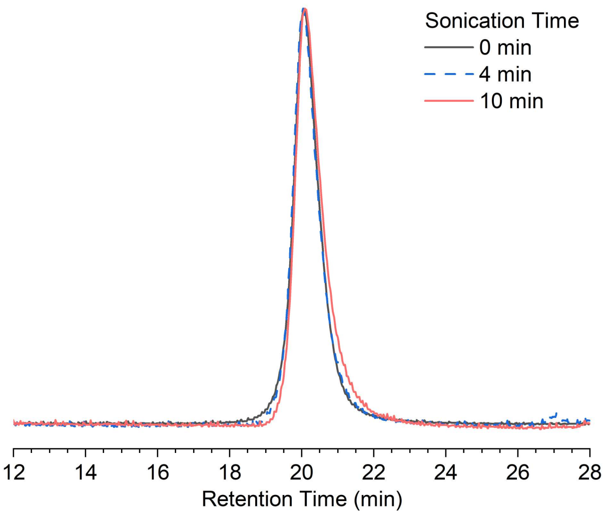

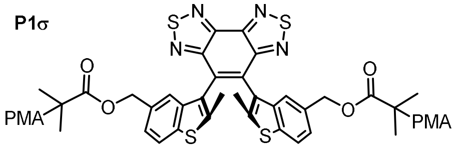

Sonication Time (min)	M_n (kDa)	M_w (kDa)	M_p (kDa)	M_w/M_n
0	84.4	100.4	100.9	1.19
4	82.5	97.4	102.5	1.18
10	72.2	87.3	97.2	1.21

Figure S7. GPC characterization of polymer **P1σ** as a function of ultrasonication time. GPC traces are baseline-corrected by subtracting straight line and then normalized by peak height.

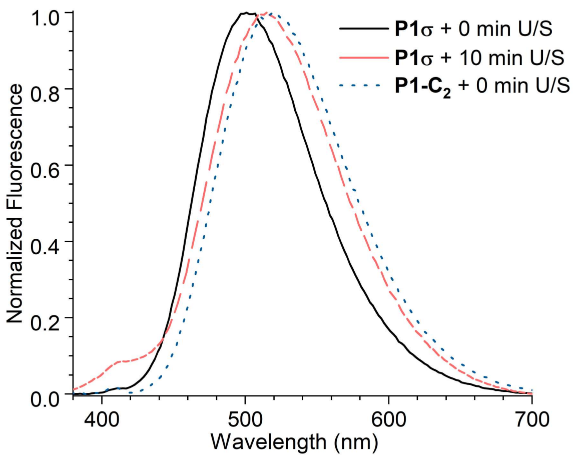

Figure S8. Normalized fluorescence emission spectra of DAE-containing polymers. U/S-induced conversion caused a bathochromic shift of the fluorescence of **P1σ** by about 15 nm (from the black solid line to the red dash line), which is consistent with the generation of anti-parallel DAEσ diastereomer (blue dotted line).

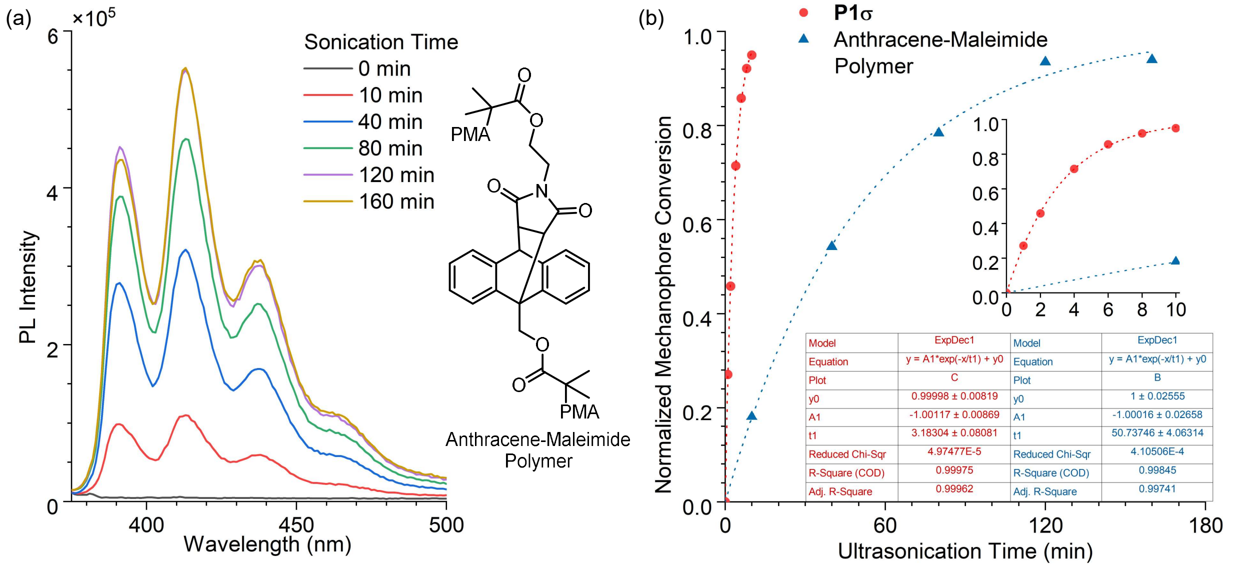

Figure S9. Comparison of the mechanosensitivity of DAEσ (F_{max} = 0.6 nN, DFT) and anthracene-maleimide (F_{max} = 4.1 nN, DFT) mechanophores. (a) shows the U/S-induced activation of an anthracene-maleimide polymer (2 mg/mL in acetonitrile) characterized by fluorescence spectroscopy (λ_{ex} = 365 nm). The anthracene-maleimide polymer (M_n = 93.6 kDa, M_p = 107.5 kDa, $Đ$ = 1.13) was synthesized following literature procedures (*J. Am. Chem. Soc.* **2013**, 135, 12722-12729). (b) summarizes the results of time-course ultrasonication experiments: The activation of DAEσ (red circle) was plotted using the OD values at 519 nm from Figure 4b. The activation of anthracene-maleimide (blue triangle) was plotted using the values of fluorescence emission at 420 nm from Figure S9a. DAEσ activates faster than the anthracene-maleimide mechanophore under identical ultrasonication conditions, supporting the high mechanosensitivity of DAEσ predicted by DFT.

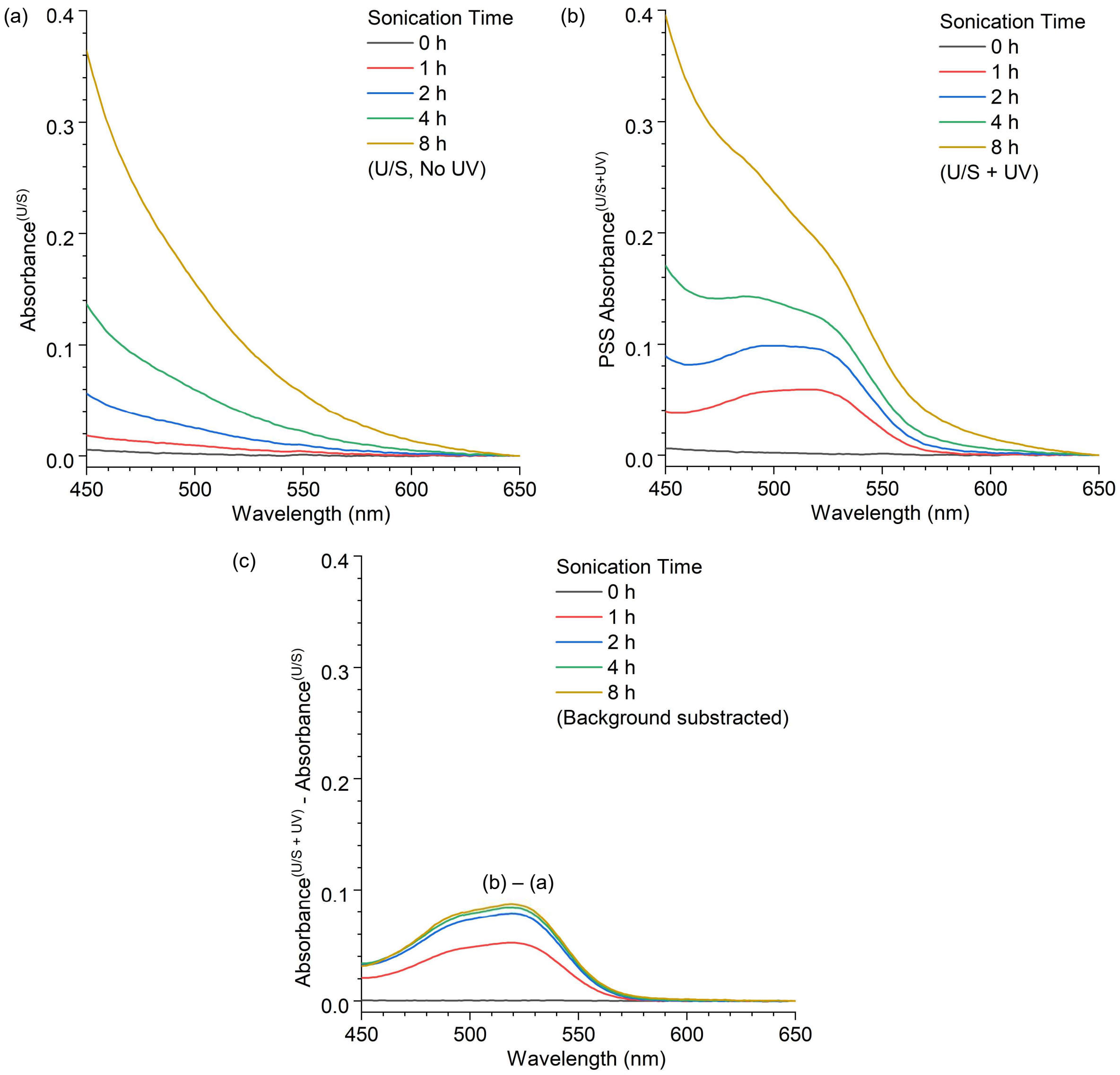

Figure S10. (a) The absorbance of a 0.5 mg/mL acetonitrile solution of **P1σ**$^{29.9k}$ (a low-MW analog of **P1σ**, M_n = 29.9 kDa, M_p = 32.5 kDa, $Đ$ = 1.15) gradually increased as byproducts accumulated over the course of prolonged ultrasonication. (b) Absorbance of ultrasonicated **P1σ**$^{29.9k}$ solutions in PSS (achieved under 365 nm) as a function of ultrasonication time. To highlight the transformation of **P1σ**$^{29.9k}$, we generated plot (c) by subtracting each line in (a) from the corresponding lines in (b) to cancel the background byproduct absorbance. (c) clearly demonstrates that DAEσ in the low-MW **P1σ**$^{29.9k}$ was successfully transformed into its photochromic parallel isomer, supporting the high mechanosensitivity of DAEσ suggested by DFT.

5. Synthetic Details

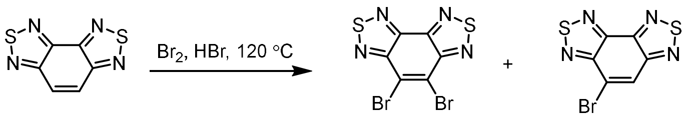

BBT (27%) 66%

4,5-Dibromobenzo[1,2-c:3,4-c′]bis[1,2,5]thiadiazole (BBT). Benzothiadiazole (0.50 g, 2.6 mmol) was suspended in 48% w/w aq. HBr (10 mL) in a 20 mL pressure reaction vessel equipped with a stir bar. Under dark conditions, bromine (0.5 mL, 9.7 mmol) was added, and the mixture was stirred at 120 °C. After two days, the reaction was cooled to room temperature, and another 0.5 mL of bromine was added. The sealed reaction was continued at 120 °C in the dark for another two days. The reaction was cooled down to room temperature followed by the addition of ice to form yellow solid precipitates, which were collected by filtration, washed with water, and washed multiple times with methanol to remove water. The resultant crude is a mixture of the monobromination and dibromination products which was carefully washed with small portions of dichloromethane (~2 mL each wash) multiple times to remove the readily soluble monobromination product, affording the desirable dibromination product **BBT** as a light-yellow solid (242 mg, 27%). ^{1}H NMR spectrum shows no **BBT** signal. ^{13}C{^{1}H} NMR (100 MHz, $CDCl_3$) δ: 155.03, 145.72, 122.46. HRMS (ESI, m/z): calcd for $[C_6HBr_2N_4S_2]^+$ $(M+H)^+$, 350.8004; found, 350.8002.

Scheme S1. Synthesis of the DAE Intermediate 6 in Isolated Atropisomeric Forms.

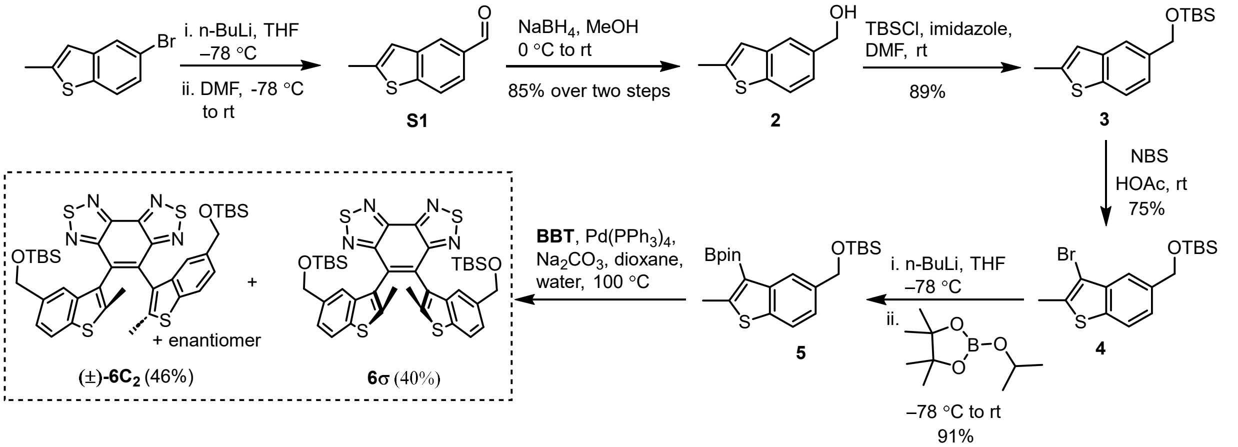

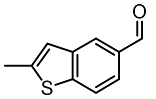

S1

2-Methylbenzo[b]thiophene-5-carboxaldehyde (**S1**). A round bottom flask equipped with a stir bar was charged with 5-bromo-2-methyl-1-benzothiophene (2.54 g, 11.19 mmol) and 40 mL anhydrous THF. The solution was cooled to −78 °C in an acetone/dry ice bath, and n-butyllithium (2.5 M in hexanes, 6.70 mL, 16.77 mmol) was added dropwise. After stirring the mixture for 30

min at −78 °C, DMF (2.59 mL, 33.55 mmol) was added to the mixture dropwise. The mixture was then allowed to warm up to room temperature for over 30 min, followed by the slow addition of 10% NH_4Cl (100 mL) to quench the reaction. The mixture was extracted with EtOAc (100 mL), and washed with brine (100 mL). The organic fraction was dried over Na_2SO_4, filtered, and concentrated under reduced pressure to yield a crude mixture. The crude product was used in the next step without further purification. Crude ^{1}H NMR (400 MHz, $CDCl_3$): 10.07 (s, 1H), 8.14 (d, J = 1.6 Hz, 1H), 7.87 (d, J = 8.3 Hz, 1H), 7.77 (dd, J = 8.3, 1.6 Hz, 1H), 7.11 (s, 1H), 2.63 (d, J = 1.2 Hz, 3H).

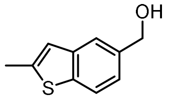

2

(2-methylbenzo[b]thiophen-5-yl)methanol (2). Crude **S1** (2.41 g) was dissolved in 20 mL ethanol at 0 °C before adding $NaBH_4$ (2.07 g, 54.7 mmol). The stirred mixture was allowed to warm up to room temperature over ~30 min. 10% NH_4Cl (20 mL) was added to the mixture dropwise to quench the reaction. The mixture was extracted by EtOAc (150 mL). The organic phase was washed with water (150 mL) and brine (150 mL), dried over Na_2SO_4, filtered, and concentrated under reduced pressure. The crude product was purified by column chromatography (10–50% EtOAC/hexanes) to provide the title compound as a white solid (1.71 g, 85% over two steps). R_f = 0.5 (EtOAc : hexane 1:5). ^{1}H NMR (400 MHz, $CDCl_3$) δ: 7.73 (d, J = 8.2 Hz, 1H), 7.65 (d, J = 1.7 Hz, 1H), 7.26 (dd, J = 8.2, 1.7 Hz, 2H), 6.96 (t, J = 1.1 Hz, 1H), 4.78 (s, 2H), 2.59 (d, J = 1.2 Hz, 3H). $^{13}C\{^1H\}$ NMR (100 MHz, $CDCl_3$) δ: 141.78, 140.84, 139.21, 137.11, 122.90, 122.28, 121.69, 121.15, 65.79, 16.36. HRMS (ESI, m/z): calcd for $[C_{10}H_9S]^+$ $(M{-}OH)^+$, 161.0419; found, 161.0420.

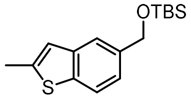

3

Tert-butyldimethyl((2-methylbenzo[b]thiophen-5-yl)methoxy)silane (3). Alcohol **2** (1.71 g, 9.6 mmol) was dissolved in DMF (10 mL) in a flame-dried round bottom flask equipped with a stir bar. Imidazole (0.654 g, 9.6 mmol) and tert-butyldimethylsilyl chloride (1.59 g, 10.55 mmol) were subsequently added to the solution. After stirring the mixture for 30 min at room temperature, 10% NH_4Cl (100 mL) was added to quench the reaction. The mixture was extracted with Et_2O, and washed with water (100 mL) and brine (100 mL). The organic phase was dried over Na_2SO_4, and concentrated under reduced pressure. The product was separated by column chromatography (0–4% EtOAc/hexanes) to yield the title compound as a colorless liquid (2.46 g, 89%). R_f = 0.4 (EtOAc:hexanes 1:50). ^{1}H NMR (400 MHz, $CDCl_3$) δ: 7.68 (d, J = 8.2 Hz, 1H), 7.60 (d, J = 1.9 Hz, 1H), 7.21 (dd, J = 8.3, 1.6 Hz, 1H), 6.98 – 6.93 (s, 1H), 4.82 (s, 2H), 2.58 (d, J = 1.2 Hz, 3H), 0.95 (s, 9H), 0.11 (s, 6H). $^{13}C\{^1H\}$ NMR (100 MHz, $CDCl_3$) δ: 141.24, 140.65, 138.45, 137.70, 122.18, 121.85, 121.79, 120.16, 65.35, 26.14, 18.62, 16.34, -5.02. HRMS (ESI, m/z): calcd for $[C_{16}H_{28}NOSSi]^+$ $(M{+}NH_4)^+$, 310.1655; found, 310.1652.

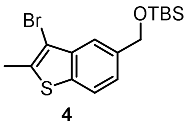

4

((3-bromo-2-methylbenzo[b]thiophen-5-yl)methoxy)(tert-butyl)dimethylsilane (4). Compound **3** (2.46 g, 8.4 mmol) was dissolved in acetic acid (50 mL) in a round bottom flask equipped with a stir bar. N-Bromosuccinimide (1.50 g, 8.4 mmol) was added into the mixture in the dark. The mixture was stirred at room temperature overnight, and concentrated under reduced pressure. The crude oil was dissolved in EtOAc (100 mL), and washed with water (100 mL) and brine (100 mL). The product was separated by column chromatography (0–4% EtOAc/hexanes) to yield the title compound as a colorless liquid (2.36 g, 75%). $R_f = 0.4$ (EtOAc:hexanes 1:20). ^{1}H NMR (400 MHz, $CDCl_3$) δ: 7.71 – 7.64 (m, 2H), 7.34 – 7.28 (m, 1H), 4.88 (s, 2H), 2.56 (s, 3H), 0.97 (s, 9H), 0.13 (s, 6H). $^{13}C\{^1H\}$ NMR (100 MHz, $CDCl_3$) δ: ass138.80, 138.61, 135.87, 135.57, 123.42, 122.08, 120.11, 106.77, 65.13, 26.12, 18.59, 15.69, -5.02. HRMS (ESI, m/z): calcd for $[C_{16}H_{27}BrNOSSi]^+$ $(M+NH_4)^+$, 388.0761; found, 388.0754.

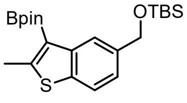

5

Tert-butyldimethyl((2-methyl-3-(4,4,5,5-tetramethyl-1,3,2-dioxaborolan-2-yl)benzo[b]thiophen-5-yl)methoxy)silane (5). A flame-dried round bottom flask equipped with a stir bar was charged with **4** (1.76 g, 4.7 mmol) and 30 mL anhydrous THF. The mixture was cooled to −78 °C in an acetone/dry ice bath. n-Butyllithium (2.5 M in hexanes, 2.28 mL, 5.7 mmol) was added dropwise. The mixture was retained at −78 °C for 30 min before adding 2-isopropoxy-4,4,5,5-tetramethyl-1,3,2-dioxaborolane (1.16 mL, 5.7 mM). The mixture was then allowed to slowly warm up to room temperature over 2.5 hours. The reaction was diluted by Et_2O (50 mL) then quenched with 10 % NH_4Cl (100 mL). The organic phase was washed with water (100 mL) and brine (100 mL), and dried over Na_2SO_4. The crude product was concentrated under reduced pressure and purified by column chromatography (25–75% DCM/hexanes) to yield the title compound as a colorless liquid (1.80 g, 91%). $R_f = 0.5$ (DCM:hexanes 2:1). ^{1}H NMR (400 MHz, $CDCl_3$) δ: 8.27 (dd, J = 1.8, 0.9 Hz, 1H), 7.67 (d, J = 8.2 Hz, 1H), 7.17 (dd, J = 8.2, 1.7 Hz, 1H), 4.87 (s, 2H), 2.80 (s, 3H), 1.38 (s, 12H), 0.98 (s, 9H), 0.14 (s, 6H). $^{13}C\{^1H\}$ NMR (100 MHz, $CDCl_3$) δ: 155.56, 144.94, 138.14, 137.62, 122.40, 121.98, 121.05, 83.18, 65.52, 26.23, 25.12, 18.62, 16.97, -4.99. HRMS (ESI, m/z): calcd for $[C_{22}H_{39}BNO_3SSi]^+$ $(M+NH_4)^+$, 436.2507; found, 46.2505.

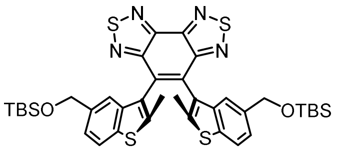

6σ

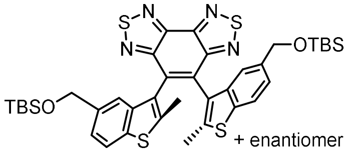

(±)-6C$_2$

Compound 6. BBT (80 mg, 0.227 mmol), compound **5** (370 mg, 0.88 mmol), tetrakis(triphenylphosphine)palladium(0) (42 mg, 0.036 mmol) were dissolved in dioxane (5 mL) in a 20 mL vial equipped with a stir bar and a rubber septum. 1 mL Na_2CO_3 (2 mmol) aqueous solution was added, then the reaction in the sealed vial was purged with N_2 for 30 min before heating up the reaction to 100 °C. The reaction was stirred in the dark for 60 hours, then diluted with EtOAc (50 mL), washed with 10 % NH_4Cl (50 mL), water (50 mL) and brine (50 mL), dried over Na_2SO_4, and concentrated under reduced pressure. The crude product was purified by column chromatography (1–10% EtOAc/hexanes) to provide pure compound **6σ** as a yellow solid (71 mg, 40%). We also obtained the anti-parallel stereoisomer **(±)-6C₂** (81 mg) which contained about 30% impurities that were difficult to remove. Compound **(±)-6C₂** was used in the following step without further purification.

6σ: R_f = 0.4 (EtOAc : hexanes 1:10). ^{1}H NMR (400 MHz, $CDCl_3$) δ: 7.59 (d, *J* = 8.3 Hz, 2H), 7.17 (dd, *J* = 8.3, 1.6 Hz, 2H), 6.95 (d, *J* = 1.7 Hz, 2H), 4.45 – 4.19 (ABq, 4H), 2.28 (s, 6H), 0.79 (s, 18H), -0.06 (s, 12H). $^{13}C\{^1H\}$ NMR (100 MHz, $CDCl_3$) δ: 156.97, 147.72, 140.79, 139.02, 137.59, 136.56, 131.23, 127.15, 122.05, 121.81, 119.57, 64.32, 25.91, 18.37, 15.84, -5.18, -5.24. HRMS (ESI, m/z): calcd for $[C_{38}H_{50}N_5O_2S_4Si_2]^+$ $(M+NH_4)^+$, 792.2380; found, 792.2370.

(±)-6C₂: R_f = 0.42 (EtOAc: hexanes 1:10). ^{1}H NMR (400 MHz, $CDCl_3$) δ: 8.05 (s, 0.7H)*, 7.80 (d, J = 8.2 Hz, 0.7H)*, 7.69 (d, J = 8.3 Hz, 2H), 7.29 (dd, J = 8.3, 1.6 Hz, 0.7H)*, 7.23 (dd, J = 8.2, 1.6 Hz, 2H), 7.06 (d, J = 1.6 Hz, 2H), 4.73 (s, 1.4H)*, 4.69 (s, 4H), 2.51 (s, 1.4H)*, 1.88 (s, 6H), 0.76 (s, 18H), -0.01 (d, J = 7.4 Hz, 4H)*, -0.04 (d, J = 6.2 Hz, 12H) [*: impurity]

Scheme S2. Synthesis of a Bifunctional Initiator 1σ Containing the Photoinert Parallel DAE Mechanophore and of an Analogous Monofunctional Control S2.

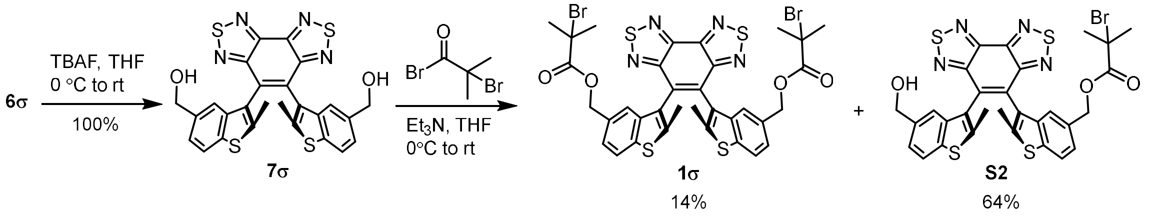

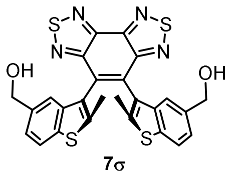
7σ

Compound 7σ. A 8 mL vial equipped with a stir bar was charged with **6σ** (130 mg, 0.092 mmol) and anhydrous THF (2 mL). The solution was cooled to 0 °C, followed by the dropwise addition of TBAF (1 M in THF, 0.20 mL, 0.20 mmol). The stirred reaction was allowed to warm up to room temperature. After one hour, the reaction was quenched with NH_4Cl (10 mL), extracted with EtOAc (50 mL), and washed with water (30 mL) and brine (30 mL). The organic fraction was dried over Na_2SO_4, filtered, and concentrated under reduced pressure. The crude product was purified by column chromatography (10–50% EtOAc/hexanes) to yield the title compound as a

yellow solid (93 mg, > 99%). R_f = 0.3 (EtOAc: hexanes 1:1). ^{1}H NMR (400 MHz, $CDCl_3$) δ: 7.58 (d, *J* = 8.2 Hz, 2H), 7.22 (d, *J* = 1.6 Hz, 2H), 7.05 (dd, *J* = 8.2, 1.6 Hz, 2H), 4.44 – 4.24 (ABq, 4H), 2.32 (s, 6H). $^{13}C\{^1H\}$ NMR (100 MHz, $CDCl_3$) δ: 156.94, 147.90, 141.14, 139.50, 137.42, 136.96, 131.28, 127.29, 123.48, 122.02, 121.29, 65.17, 15.90. HRMS (ESI, m/z): calcd for $[C_{26}H_{22}N_5O_2S_4]^+$ $(M+NH_4)^+$, 564.0651; found, 564.0638.

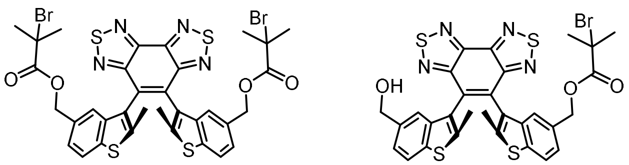

1σ S2

Compound 1σ and S2. A 8 mL vial equipped with a stir bar was charged with **7σ** (67 mg, 0.123 mmol), Et_3N (19.84 mg, 0.196 mmol), and anhydrous THF (2 mL) at 0 °C. A 1 mL THF solution of α-bromoisobutyryl bromide (45 mg, 0.196 mmol) was added into the reaction dropwise. After overnight reaction at room temperature, the reaction was quenched by the addition of 10% NH_4Cl (10 mL) and extracted with EtOAc (50 mL). The organic layer was washed with water (30 mL) and brine (30 mL), dried over Na_2SO_4, and concentrated under reduced pressure. The crude product was purified by column chromatography (15–50% EtOAc/hexanes) to yield **1σ** (13 mg, 14%) and **S2** (53 mg, 64%) as yellow solids in isolated forms.

1σ: R_f = 0.7 (EtOAc : hexanes 1:2). ^{1}H NMR (400 MHz, $CDCl_3$) δ: 7.63 (d, *J* = 8.2 Hz, 2H), 7.16 (dd, *J* = 8.3, 1.6 Hz, 2H), 7.08 (d, *J* = 1.7 Hz, 2H), 4.88 – 4.75 (ABq, 4H), 2.28 (s, 6H), 1.82 (s, 12H). $^{13}C\{^1H\}$ NMR (100 MHz, $CDCl_3$) δ: 171.30, 156.82, 147.84, 141.63, 139.14, 137.99, 131.62, 130.97, 127.26, 123.72, 122.33, 122.06, 67.20, 55.96, 30.84, 15.90. HRMS (ESI, m/z): calcd for $[C_{34}H_{28}Br_2N_4O_4S_4]^+$ $(M)^+$, 841.9355; found, 841.9342.

S2: R_f = 0.35 (EtOAc : hexanes 1:2). ^{1}H NMR (400 MHz, $CDCl_3$) δ: 7.63 (dd, J = 15.2, 8.3 Hz, 2H), 7.17 (td, J = 8.4, 1.6 Hz, 2H), 7.09 (dd, J = 19.2, 1.6 Hz, 2H), 4.92 – 4.69 (m, 2H), 4.34 (qd, J = 12.8, 5.6 Hz, 2H), 2.28 (d, J = 5.7 Hz, 6H). $^{13}C\{^1H\}$ NMR (100 MHz, $CDCl_3$) δ: 171.87, 156.88, 156.85, 147.88, 147.86, 141.86, 141.24, 139.29, 139.20, 138.16, 137.47, 137.31, 131.23, 131.12, 130.79, 127.28, 123.71, 123.58, 122.40, 122.31, 122.18, 120.93, 67.49, 65.17, 55.86, 30.75, 15.94, 15.88. HRMS (ESI, m/z): calcd for $[C_{30}H_{27}BrN_5O_3S_4]^+$ $(M+NH_4)^+$, 712.0175; found, 712.0167.

Scheme S3. Synthesis of a Bifunctional Initiator (±)-1C₂ Containing the Photoactive Anti-Parallel DAE.*

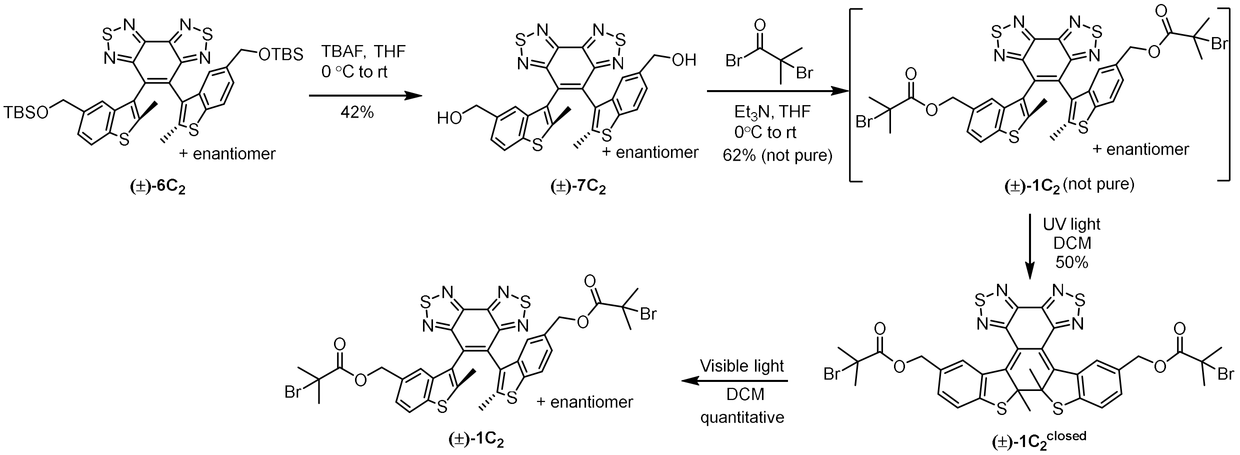

*: The **(±)-1C$_2$** prepared from the esterification reaction was difficult to purify. We found that the ring-closed **(±)-1C$_2^{closed}$** could be readily isolated. Thus, we first performed UV irradiation on the crude **(±)-1C$_2$**, isolated the ring-closed **(±)-1C$_2^{closed}$**, which was then cleanly converted to **(±)-1C$_2$** under visible light.

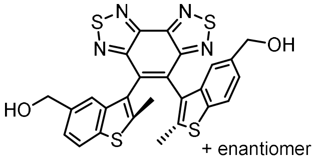

(±)-7C$_2$

Compound (±)-7C$_2$. Crude **(±)-6C$_2$** (77 mg, 0.099 mmol) was dissolved in 2mL THF followed by the dropwise addition of TBAF (1 M in THF, 0.22 mL, 0.22 mmol) at 0 °C. The reaction was stirred at room temperature for 1 h, then quenched with NH_4Cl (10 mL), extracted with EtOAc (50 mL), washed with water (30 mL) and brine (30 mL), dried over Na_2SO_4, filtered, and concentrated under reduced pressure. The crude product was purified by column chromatography (10–50% EtOAc/hexanes) to afford the title compound as a light orange solid (23 mg, 42%). R_f = 0.55 (EtOAc : hexanes 1:1). ^{1}H NMR (400 MHz, $CDCl_3$) δ: 7.63 (d, J = 8.1 Hz, 2H), 7.22 – 7.16 (m, 2H), 7.11 (s, 2H), 4.63 (s, 4H), 2.17 (s, 6H). $^{13}C\{^1H\}$ NMR (100 MHz, $CDCl_3$) δ: 157.01, 147.84, 141.60, 139.35, 137.54, 136.86, 131.29, 127.06, 123.26, 122.31, 120.76, 65.59, 16.32. HRMS (ESI, m/z) : calcd for $[C_{26}H_{22}N_5O_2S_4]^+$ $(M+NH_4)^+$, 564.0651; found, 564.0639.

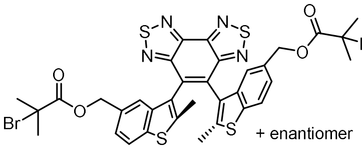

(±)-1C$_2$

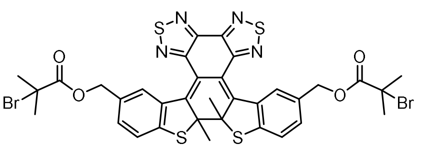

(±)-1C$_2^{closed}$

Compound (±)-1C$_2$ and **(±)-1C$_2^{closed}$**. A flame-dried 20 mL vial equipped with a stir bar and septum was charged with ± **7-C$_2$** (this batch of ± **7-C$_2$** starting material contains about 10% isomer

7σ) (105 mg, 0.192 mM), 1 mL anhydrous THF, and Et_3N (43 mg, 0.42 mmol). The solution was cooled to 0 °C in an ice bath followed by the addition of a THF solution of α-bromoisobutyryl bromide (97 mg, 0.42 mmol) dropwise. After string overnight, the reaction was concentrated and purified by column chromatography (5–30% EtOAc/hexanes) to yield **(±)-1C₂** (100mg, crude yield 62%). Unfortunately, the obtained product contains some byproduct (mainly due to the contaminant **7σ** in this batch of starting material) that were difficult to isolate.

The crude **(±)-**$\mathbf{1C_2}$ was then dissolved in 10 mL DCM in a 20 mL vial, and the solution was vigorously stirred and irradiated under 365 nm using a handheld UV lamp. After three hours of UV irradiation, the deep red solution was concentrated and purified by column chromatography (5–20% Acetone/hexanes) in the dark to yield the purified **(±)-**$\mathbf{1C_2^{closed}}$ as a red solid (50 mg, ring-closing reaction yield = 50%). ^{1}H NMR (400 MHz, $CDCl_3$) δ: 8.07 (d, *J* = 1.5 Hz, 2H), 7.34 – 7.27 (m, 4H), 5.14 – 5.05 (ABq, 4H), 2.01 (s, 6H), 1.94 (d, *J* = 6.5 Hz, 12H). ^{13}C{^{1}H} (100 MHz, $CDCl_3$) δ: 171.51, 155.75, 150.88, 148.51, 147.65, 132.26, 132.02, 131.10, 128.18, 123.90, 119.56, 67.46, 67.31, 55.75, 30.87, 24.73.

The isolated **(±)-**$\mathbf{1C_2^{closed}}$ (45 mg) was dissolved in 1-2 mL $CDCl_3$ in an NMR tube, and this solution was irradiated with visible light with a flashlight. After 30 min, ^{1}H NMR results showed complete and clean conversion of **(±)-**$\mathbf{1C_2^{closed}}$ to the ring-open isomer **(±)-**$\mathbf{1C_2}$. The solution was concentrated under reduced pressure to afford **(±)-**$\mathbf{1C_2}$ as a light red solid (45 mg, quantitative). ^{1}H NMR (400 MHz, $CDCl_3$) δ: 7.73 (d, *J* = 8.2 Hz, 2H), 7.28 (dd, *J* = 8.3, 1.7 Hz, 2H), 7.17 (s, 2H), 5.27 – 5.02 (ABq, 4H), 1.96 (s, 6H), 1.83 (s, 12H). ^{13}C{^{1}H} NMR (100 MHz, $CDCl_3$) δ: 171.31, 156.90, 147.81, 141.65, 139.68, 138.27, 131.46, 131.39, 126.81, 124.26, 122.60, 122.53, 67.71, 55.86, 30.80, 30.78, 16.05. HRMS (ESI, m/z): calcd for $[C_{34}H_{28}Br_2N_4O_4S_4]^+$ $(M+NH_4)^+$, 859.9698; found, 859.9691

General Polymerization Procedures

A 10 mL Schlenk flask equipped with a stir bar was charged with the initiator (1 equiv), methyl acrylate (~1,200 equiv), Me_6TREN (2 equiv), and DMSO (equal volume to methyl acrylate). The solution was deoxygenated via three freeze-pump-thaw cycles, and then backfilled with nitrogen. The flask was opened briefly and freshly cut copper wire (1.0 cm length, 20 gauge) was added to the frozen mixture under a blanket of nitrogen. Then, the solution was deoxygenated again under vacuum and backfilled with nitrogen. After stirring at room temperature for 35 min, the flask was opened to air and the solution was diluted with DCM. The polymer solution was precipitated into cold methanol (3x) and the isolated polymer was dried thoroughly under vacuum.

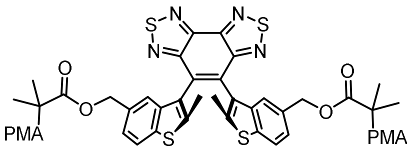

P1σ

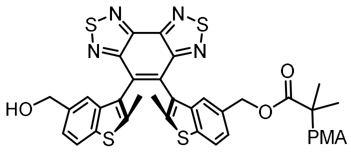

P2

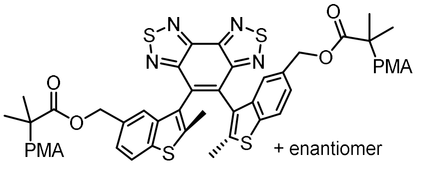

P1-C$_2$

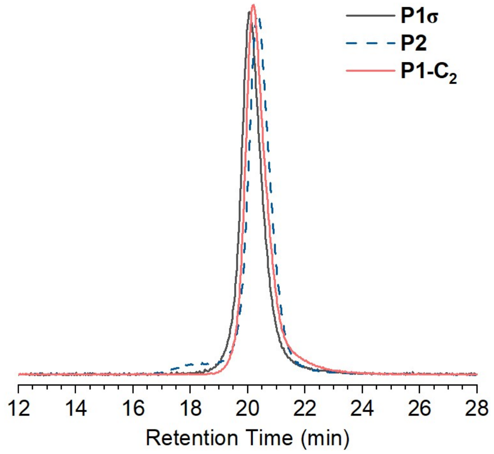

Sample	M_n (kDa)	M_w (kDa)	M_p (kDa)	M_w/M_n
**P1σ**	84.4	100.4	100.9	1.19
**P2**	70.1	120.7	78.7	1.72
**P1-C$_2$**	67.0	82.3	90.0	1.23

Figure S11. GPC traces and molecular weight data for some polymers reported in this paper.

6. NMR Spectra

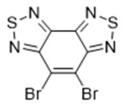

BBT

^{13}C (100 MHz, $CDCl_3$)

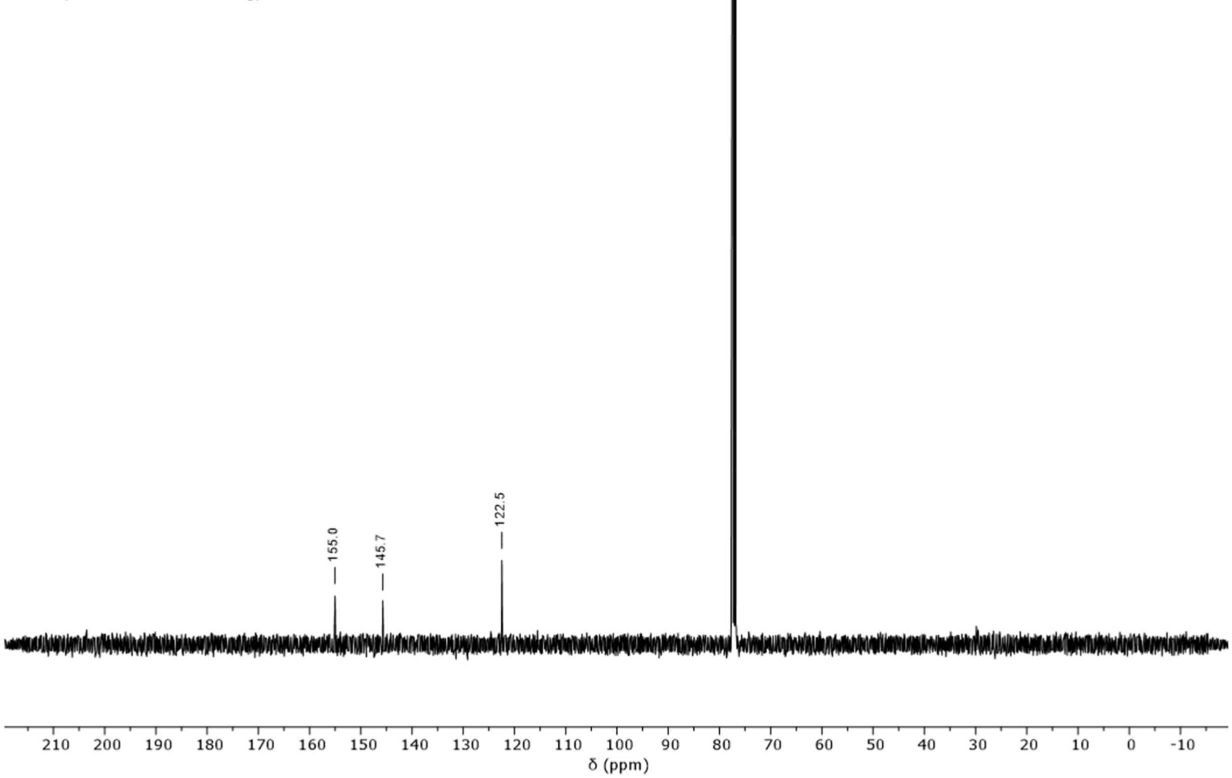

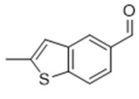

S1

^{1}H (400 MHz, $CDCl_3$)

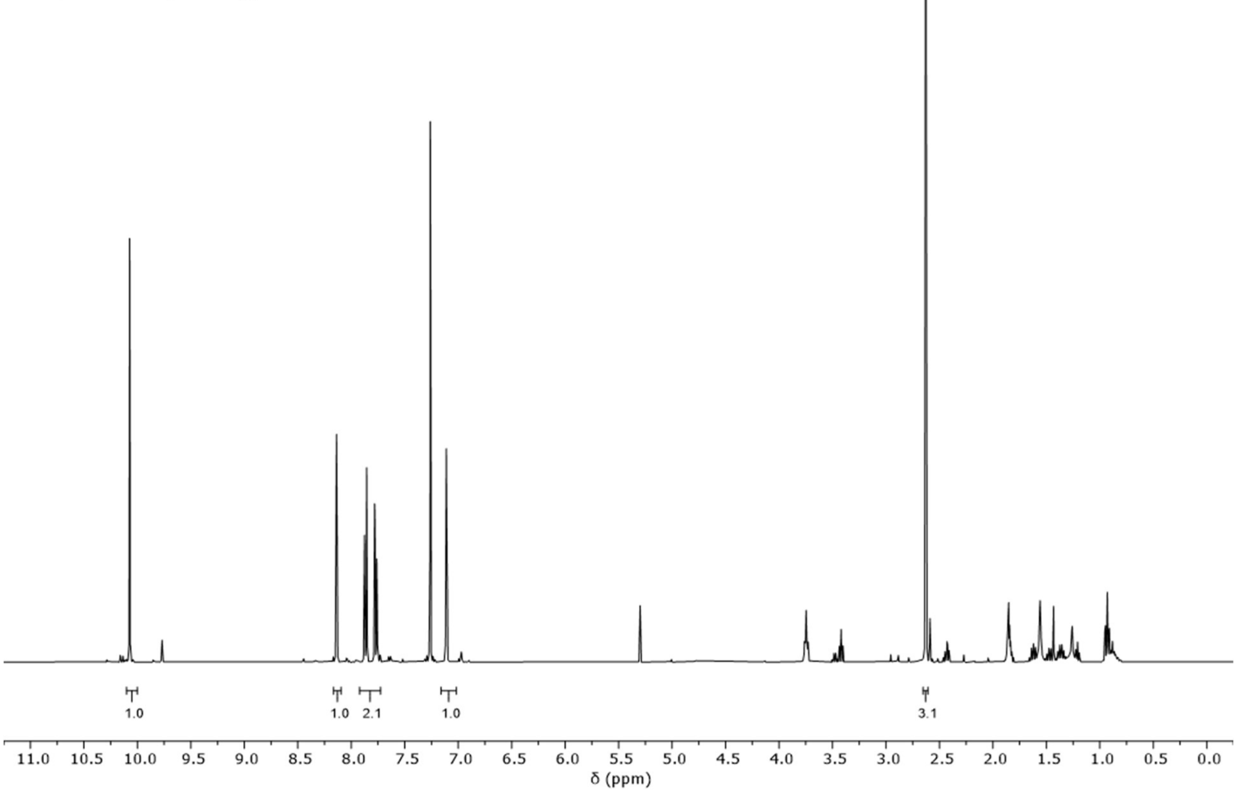

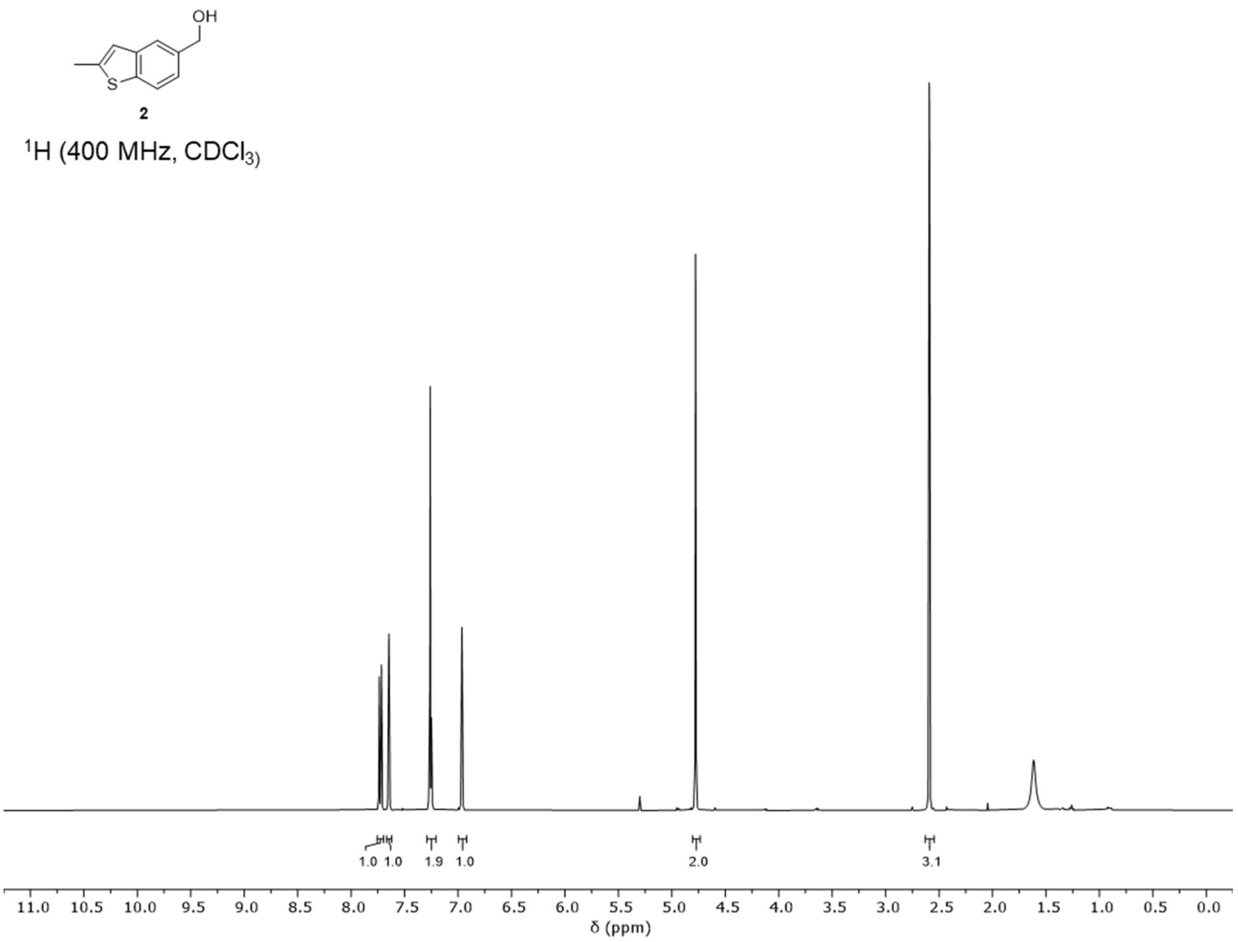
OH
S
2
1H (400 MHz, CDCl3)
1.0 1.0 1.9 1.0 2.0 3.1
11.0 10.5 10.0 9.5 9.0 8.5 8.0 7.5 7.0 6.5 6.0 5.5 5.0 4.5 4.0 3.5 3.0 2.5 2.0 1.5 1.0 0.5 0.0
δ (ppm)

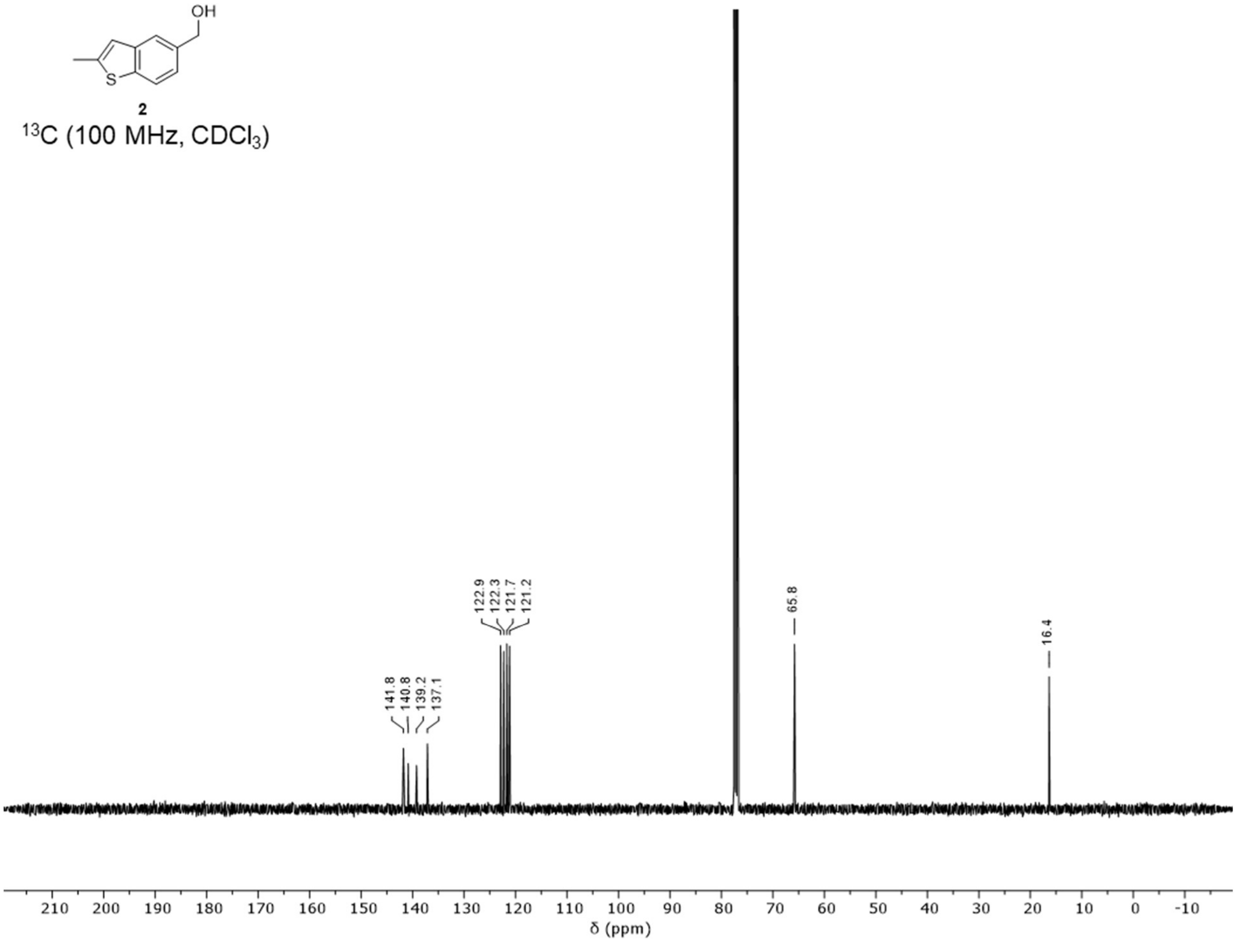
OH
S
2
13C (100 MHz, CDCl3)
141.8
140.8
139.2
137.1
122.9
122.3
121.7
121.2
65.8
16.4
210 200 190 180 170 160 150 140 130 120 110 100 90 80 70 60 50 40 30 20 10 0 -10
δ (ppm)

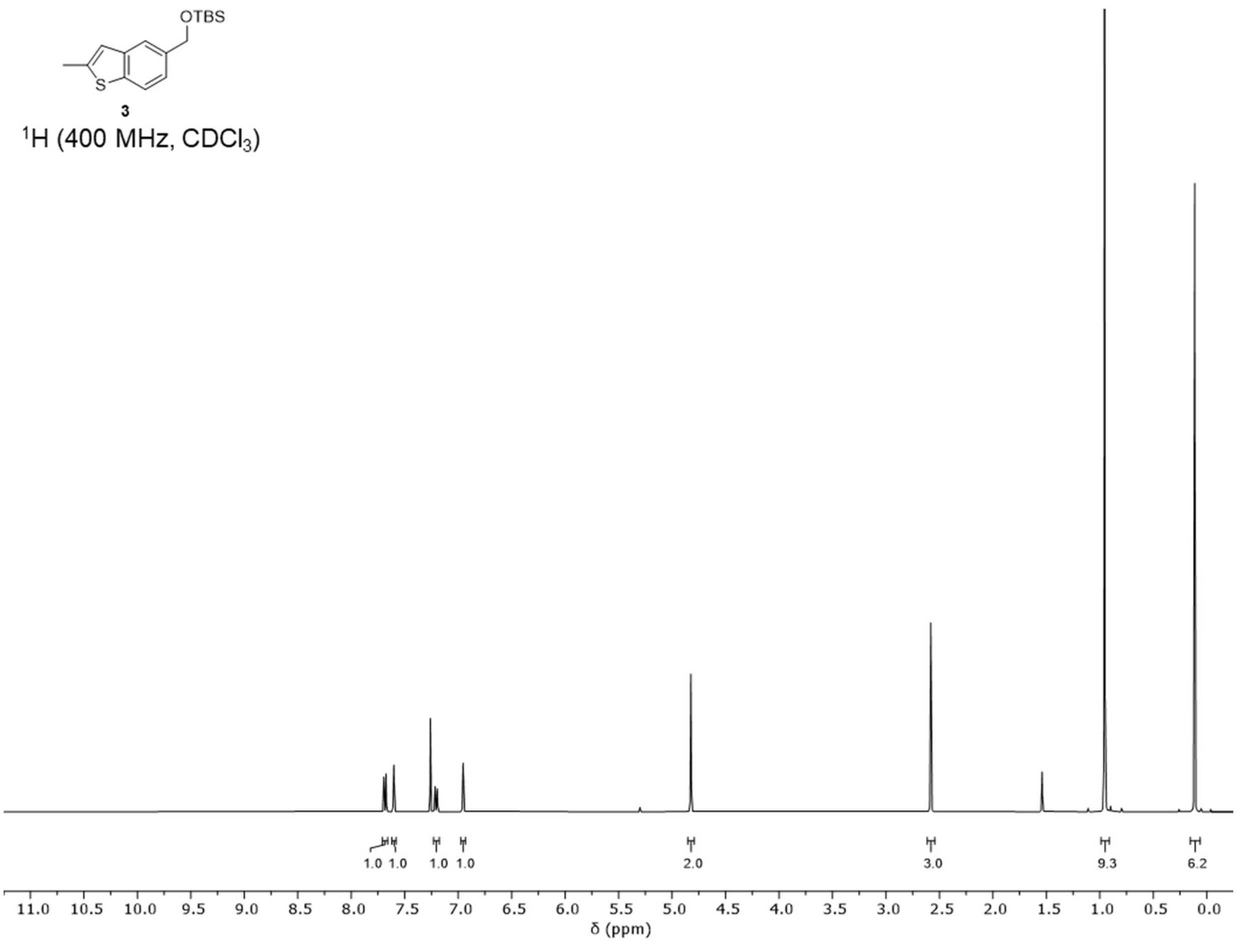

3

^{1}H (400 MHz, $CDCl_3$)

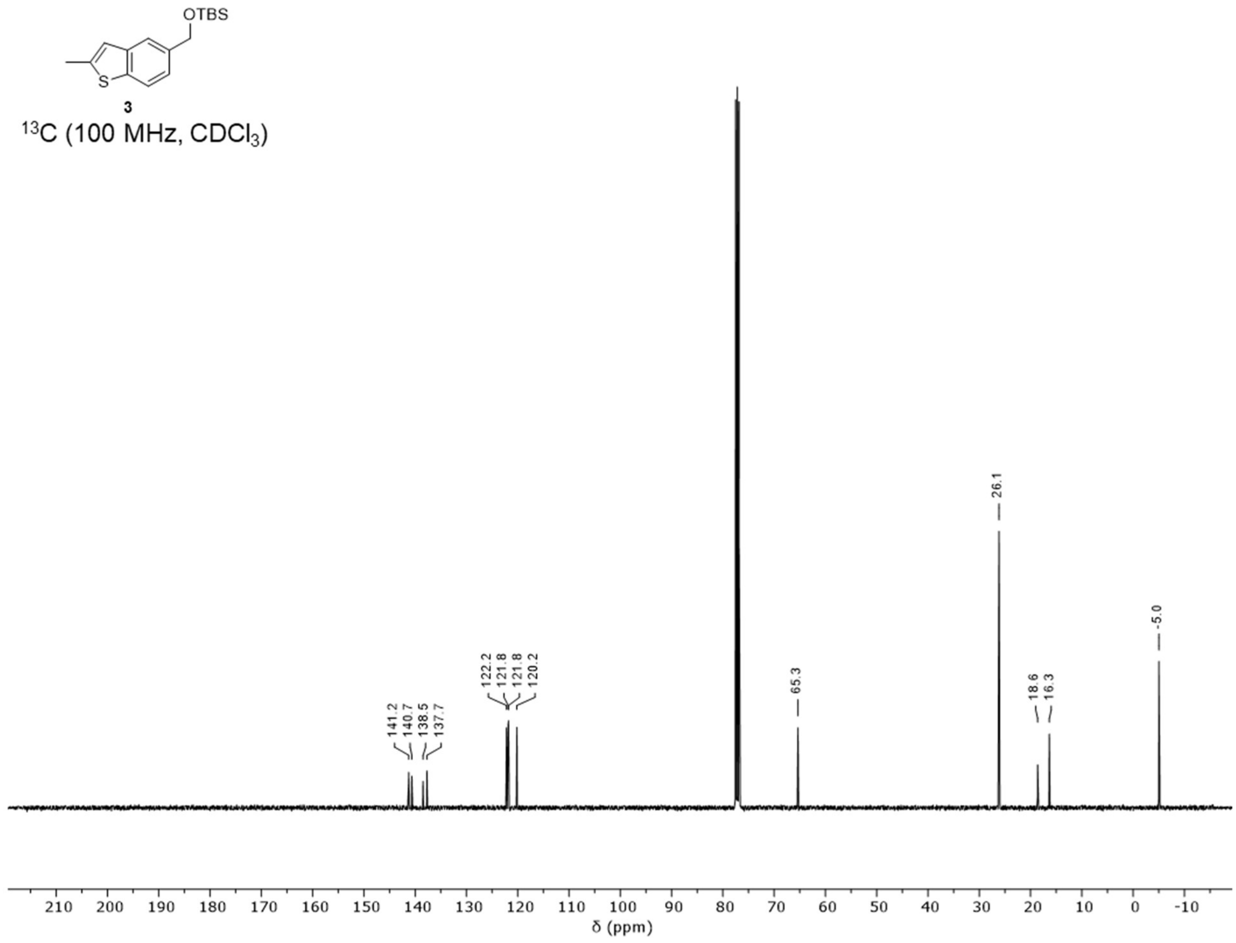

3

^{13}C (100 MHz, $CDCl_3$)

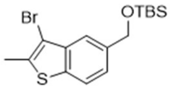

4

^{1}H (400 MHz, $CDCl_3$)

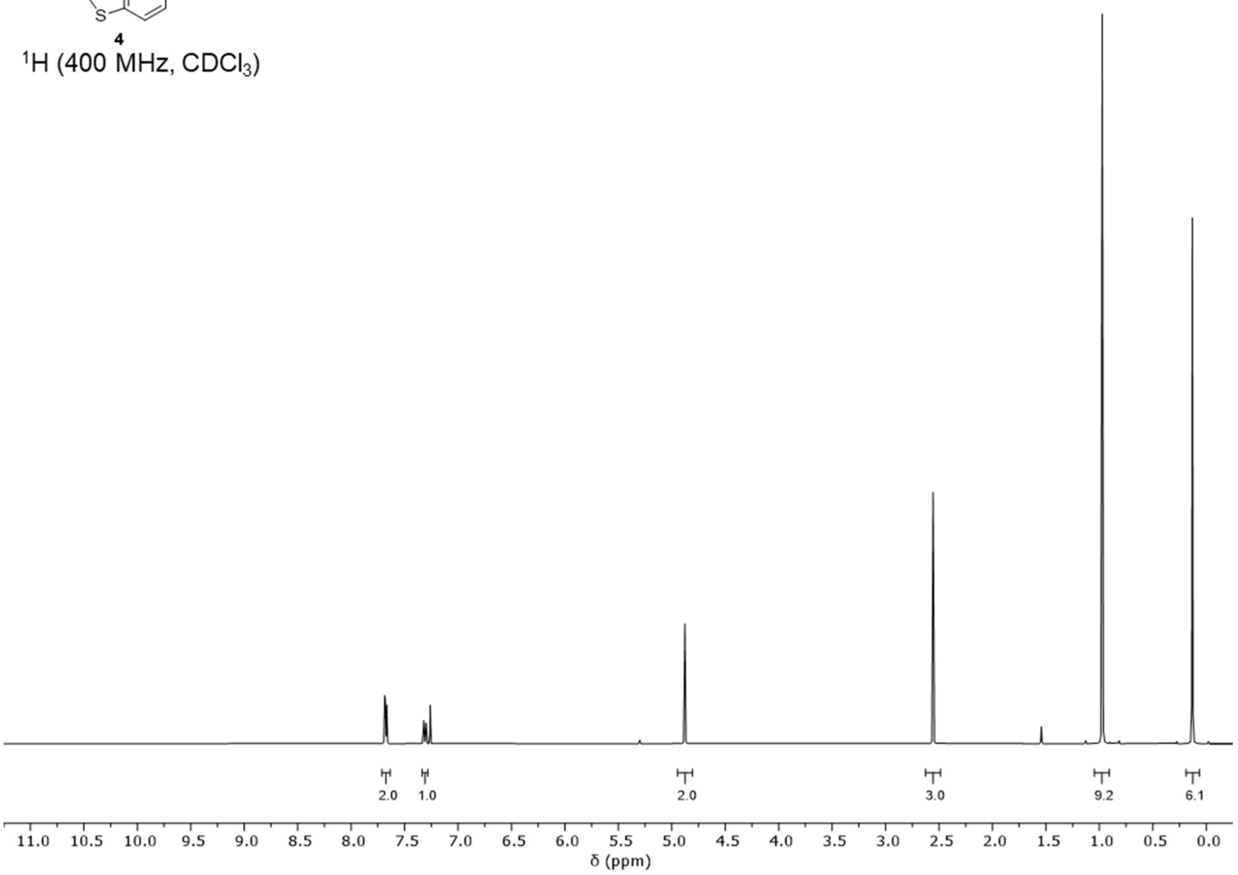

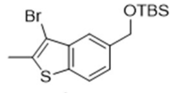

4

^{13}C (100 MHz, $CDCl_3$)

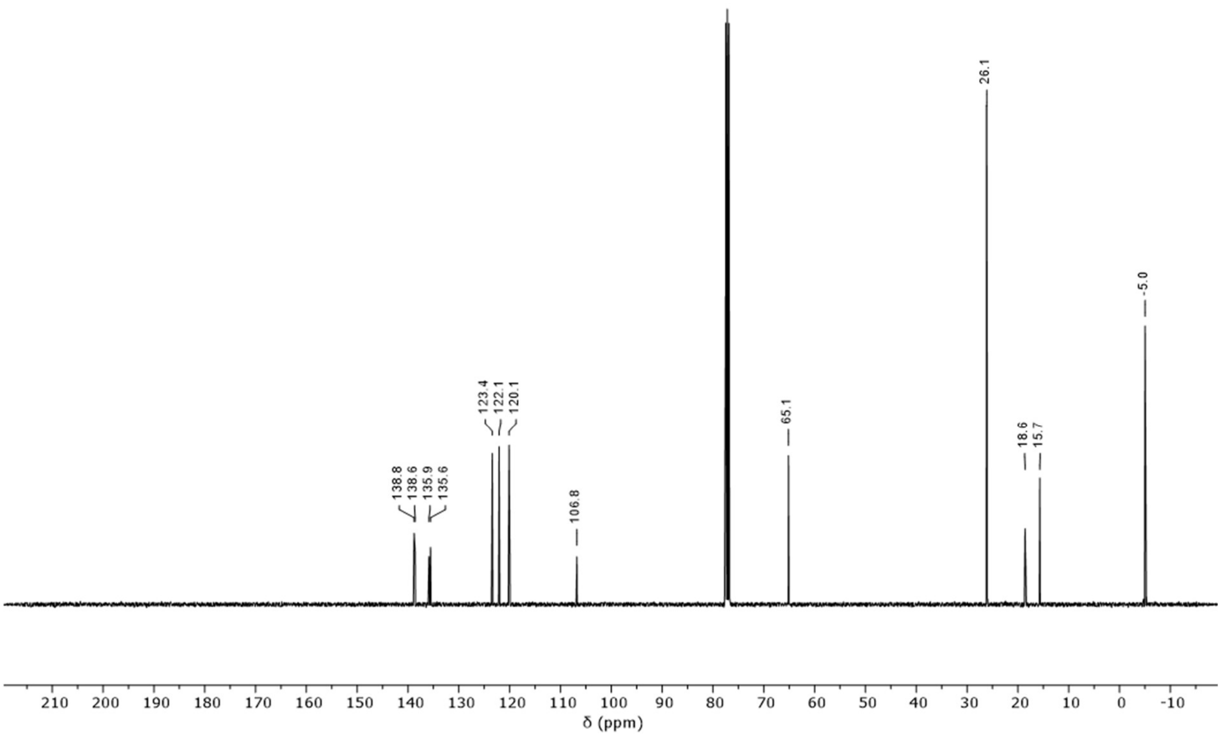

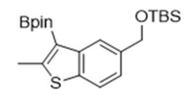

5

^{1}H (400 MHz, $CDCl_3$)

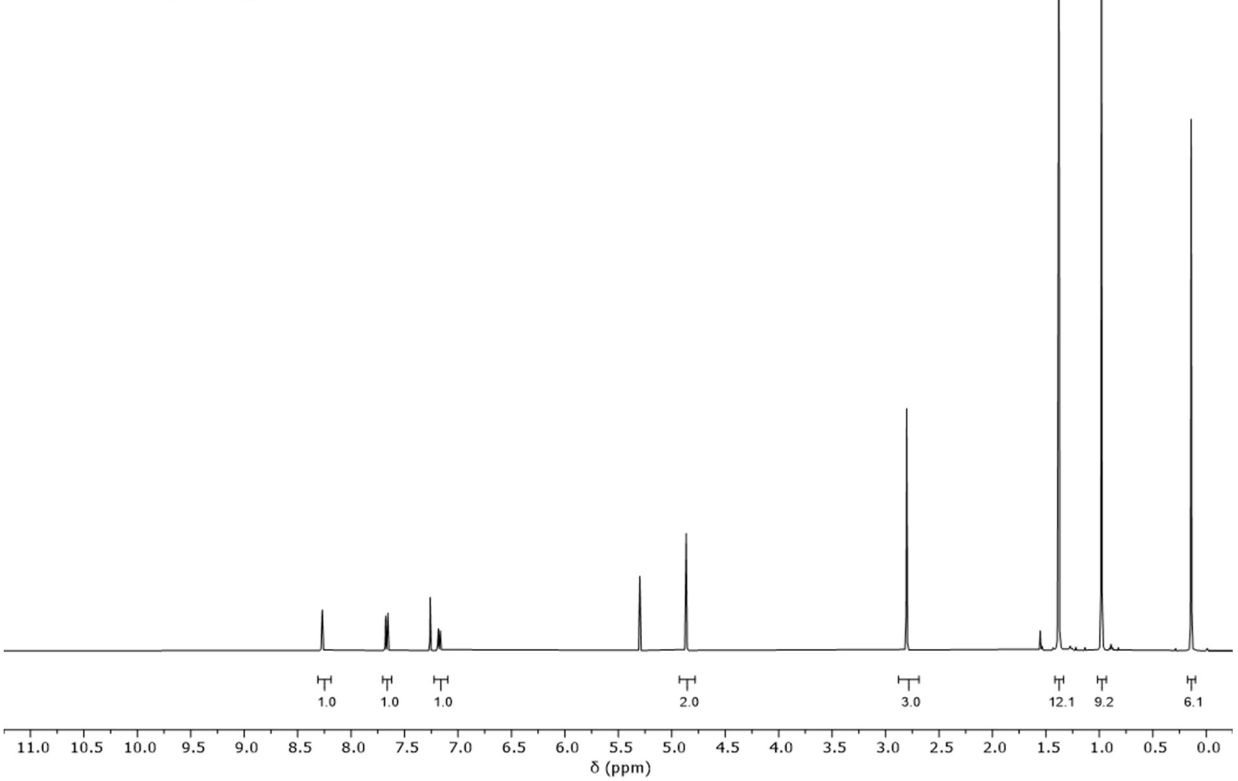

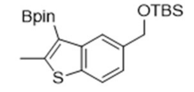

5

^{13}C (100 MHz, $CDCl_3$)

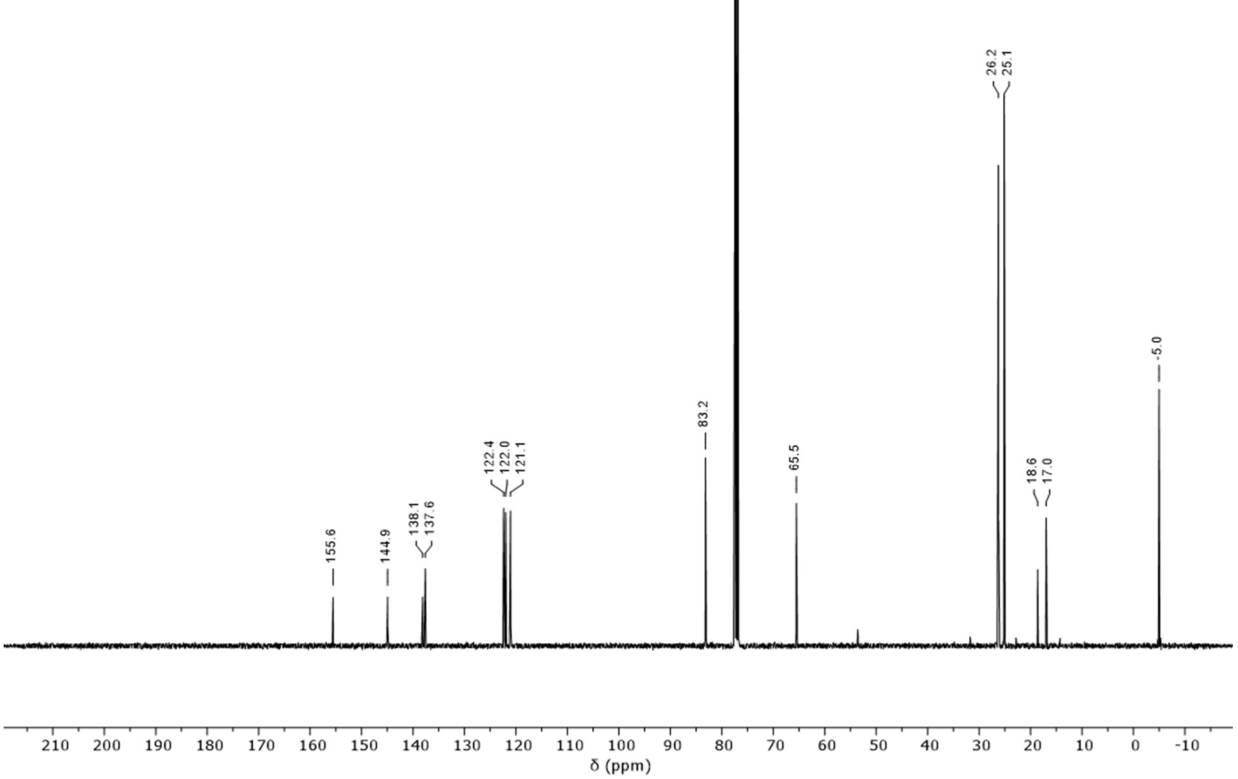

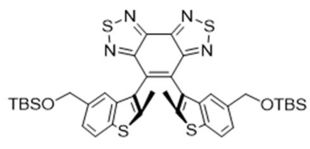

6σ

^{1}H (400 MHz, $CDCl_3$)

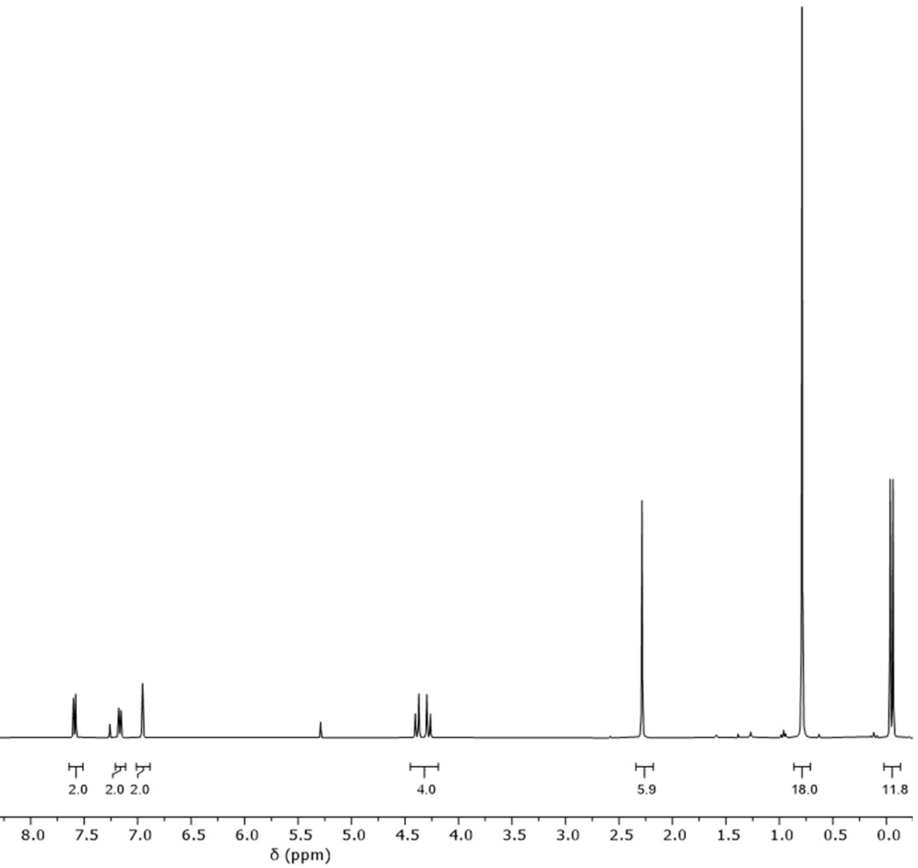

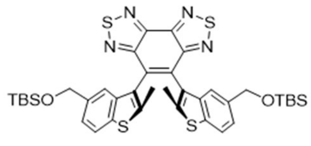

6σ

^{13}C (100 MHz, $CDCl_3$)

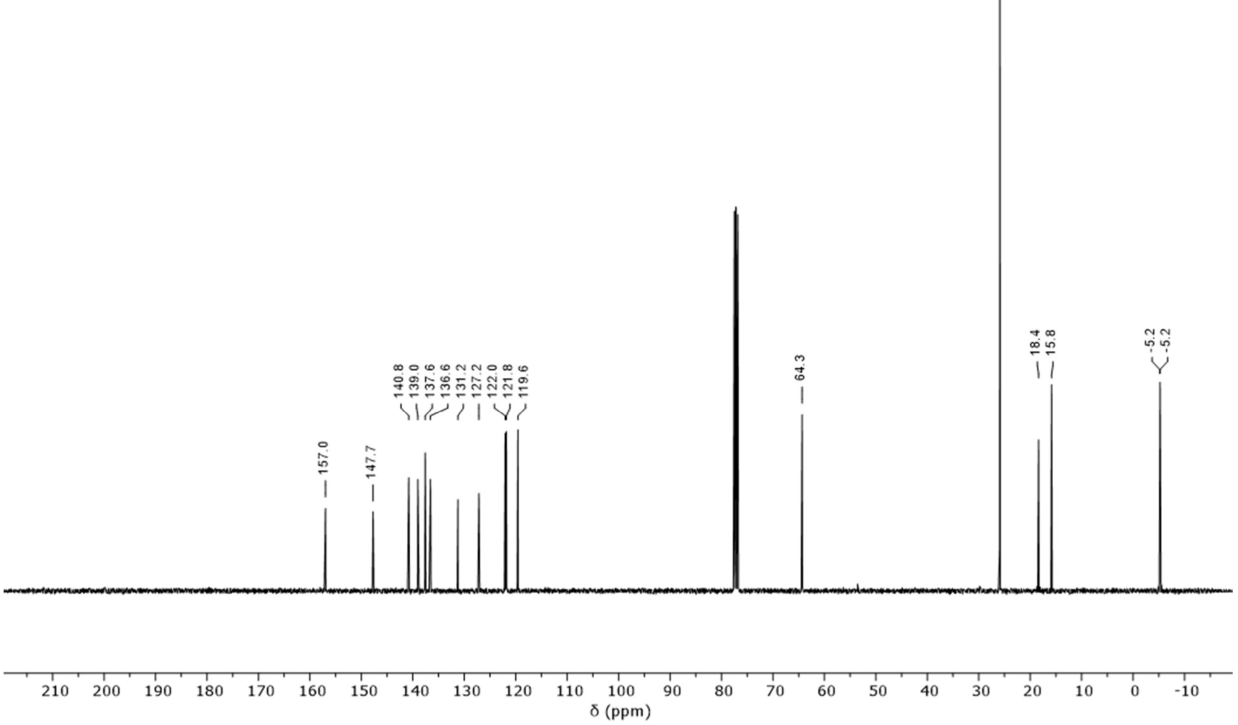

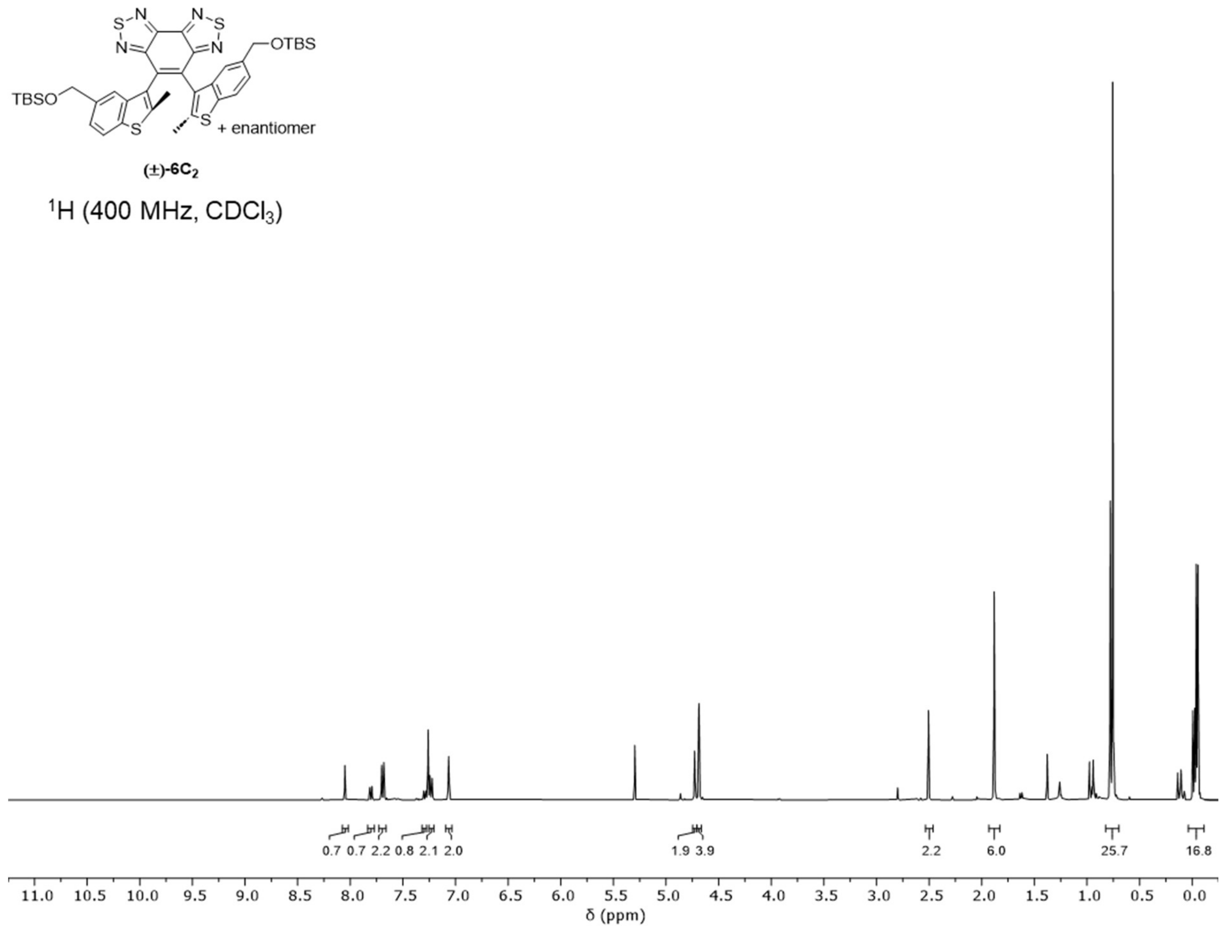

(±)-6C$_2$ contains impurities [*: impurity peaks]. We attribute the major impurity as the mono-substitution byproduct. ^{1}H NMR (400 MHz, $CDCl_3$) δ: 8.05 (s, 0.7H)*, 7.80 (d, J = 8.2 Hz, 0.7H)*, 7.69 (d, J = 8.3 Hz, 2H), 7.29 (dd, J = 8.3, 1.6 Hz, 0.7H)*, 7.23 (dd, J = 8.2, 1.6 Hz, 2H), 7.06 (d, J = 1.6 Hz, 2H), 4.73 (s, 1.4H)*, 4.69 (s, 4H), 2.51 (s, 2.2H)*, 1.88 (s, 6H), 0.78 (s, 7.3H)*, 0.76 (s, 18H), -0.01 (d, J = 7.4 Hz, 4H)*, -0.04 (d, J = 6.2 Hz, 12H)

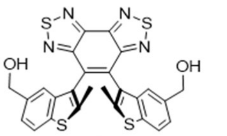

7σ

^{1}H (400 MHz, $CDCl_3$)

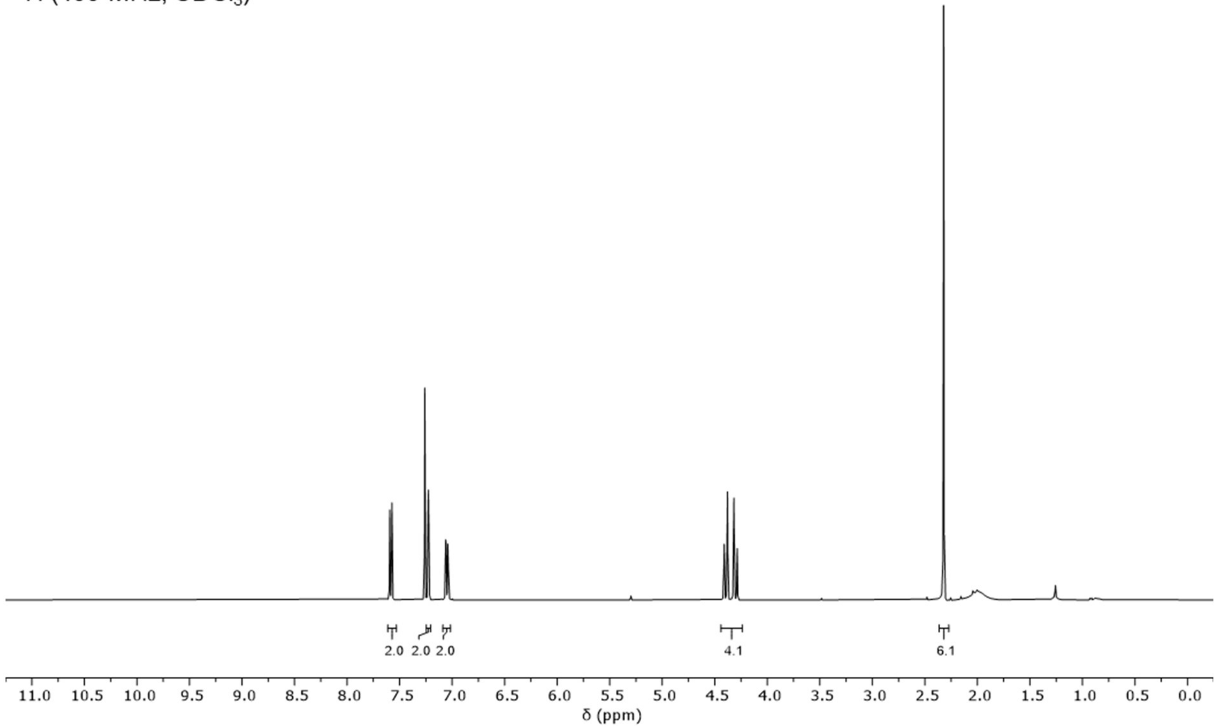

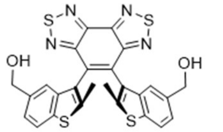

7σ

^{13}C (100 MHz, $CDCl_3$)

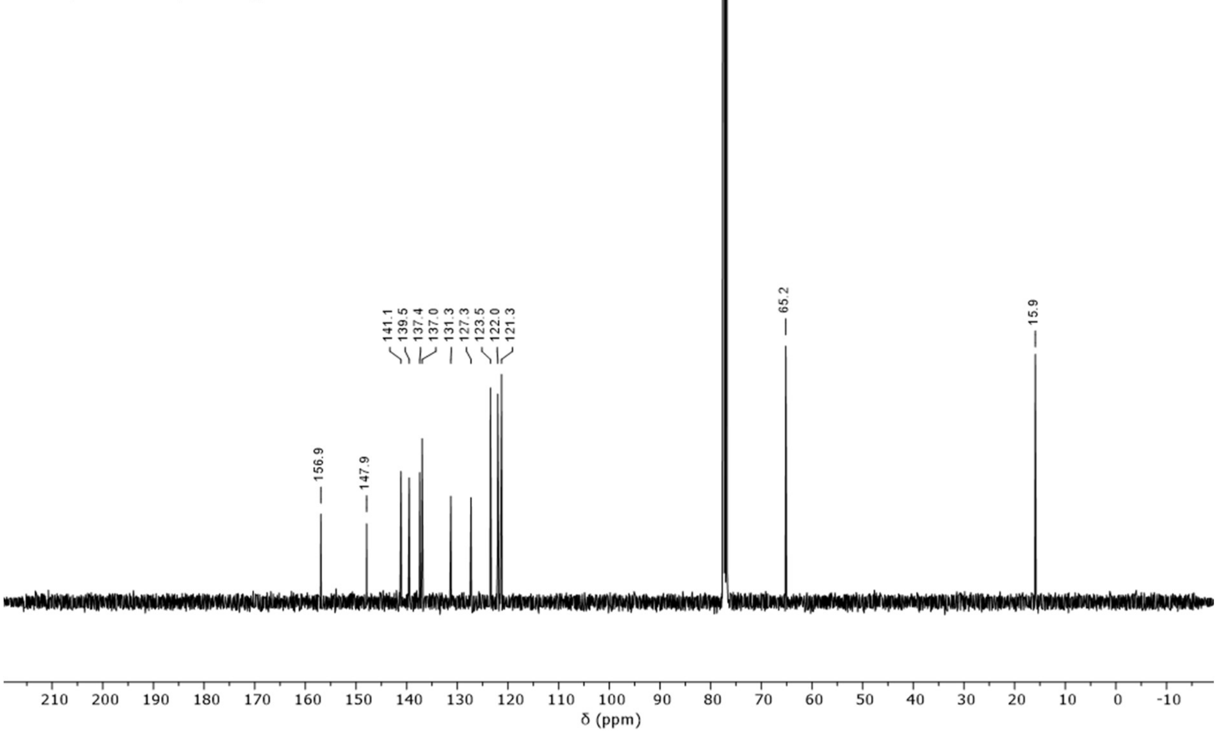

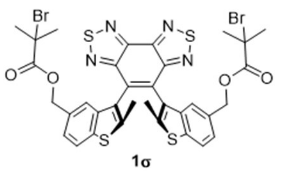

1σ

^{1}H (400 MHz, $CDCl_3$)

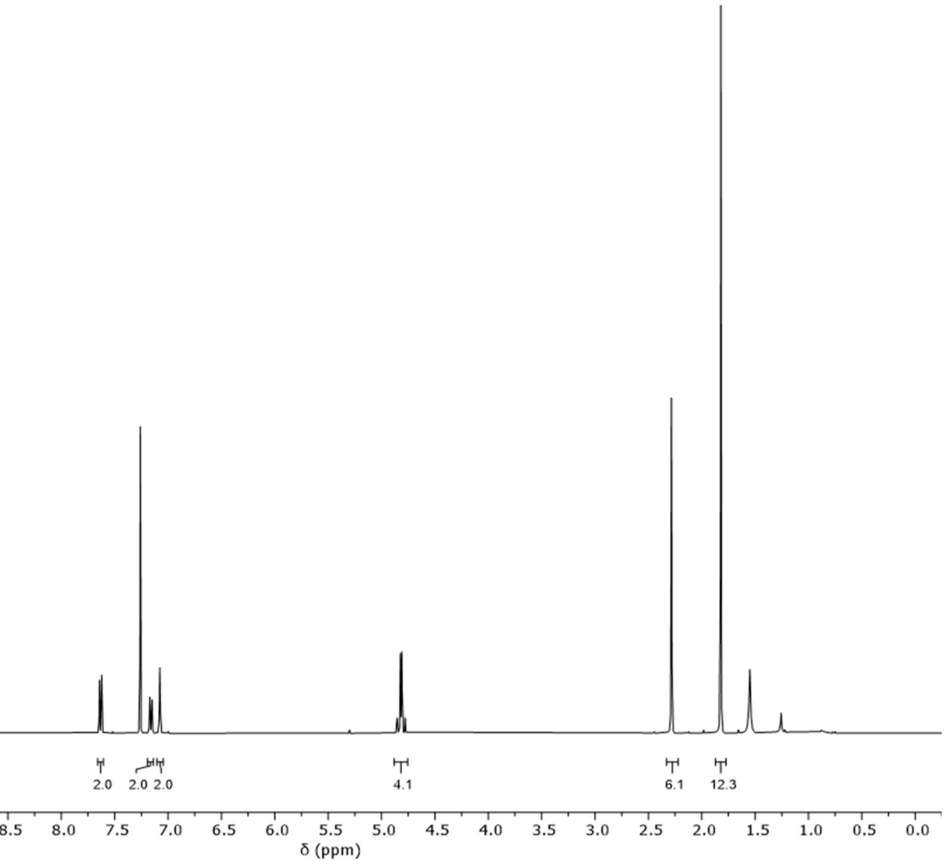

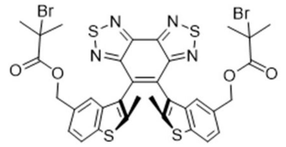

1σ

^{13}C (100 MHz, $CDCl_3$)

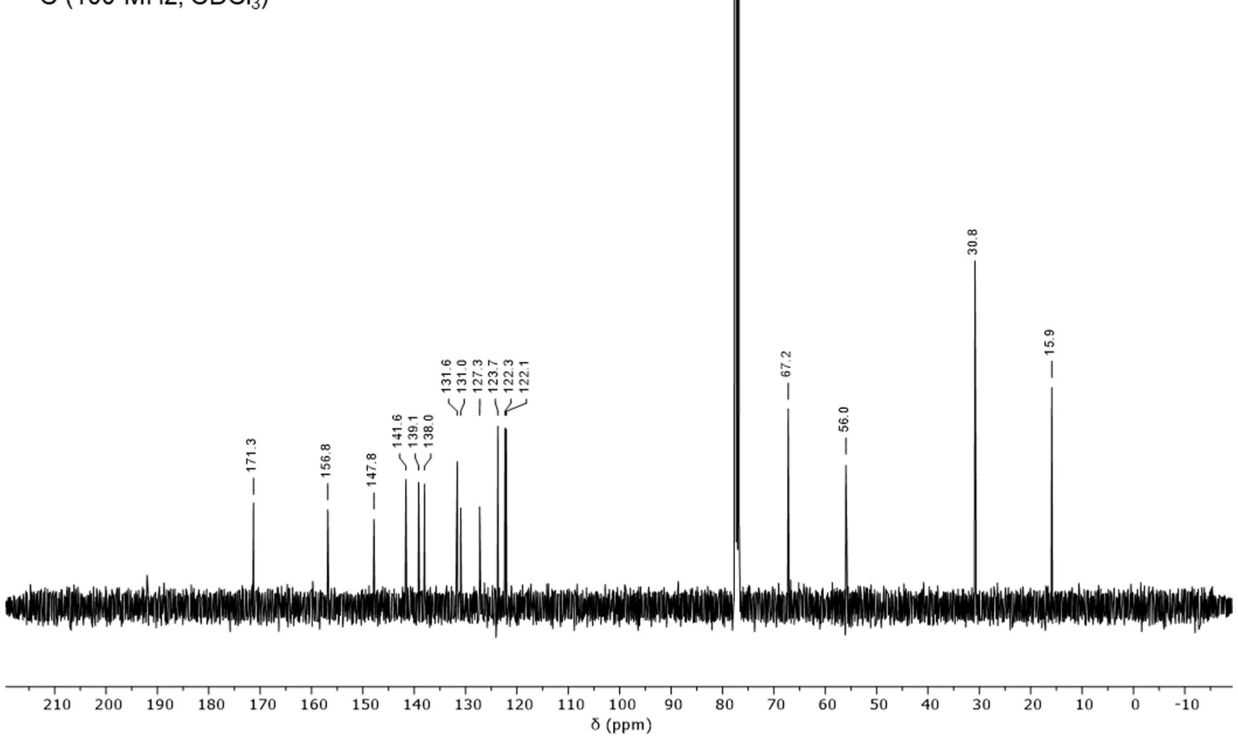

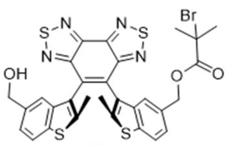

S2

^{1}H (400 MHz, $CDCl_3$)

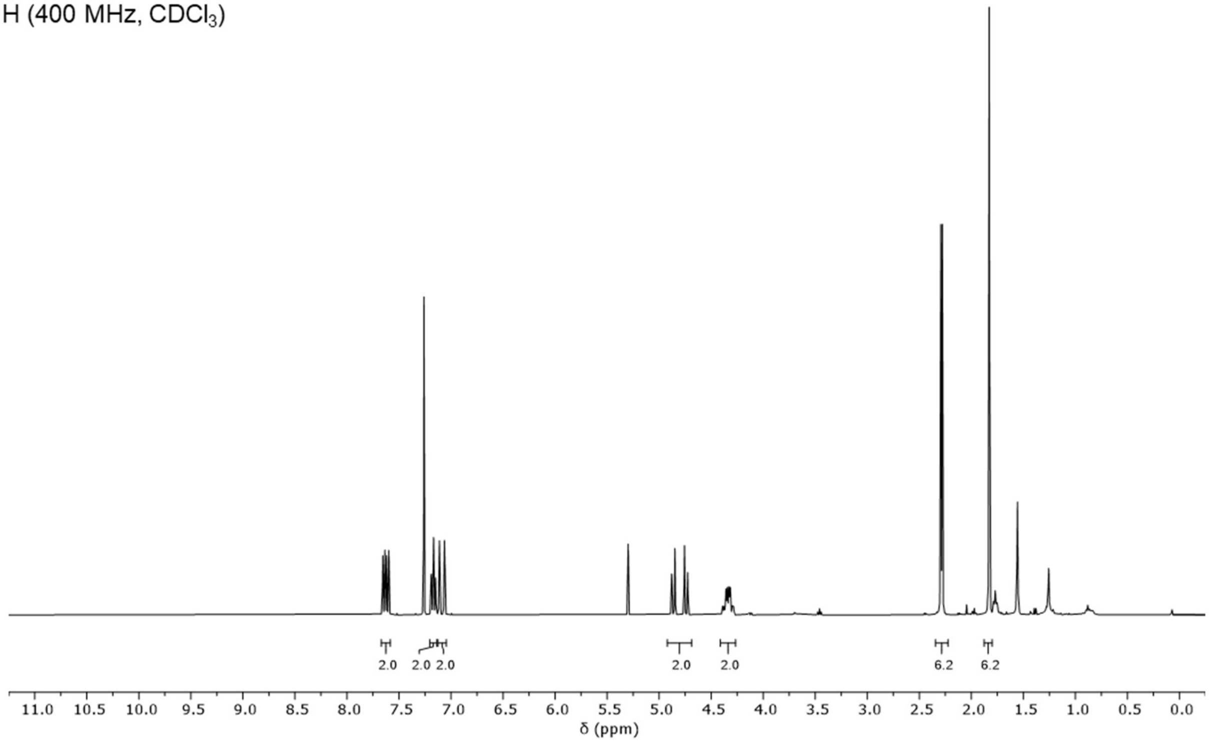

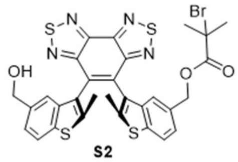

S2

^{13}C (100 MHz, $CDCl_3$)

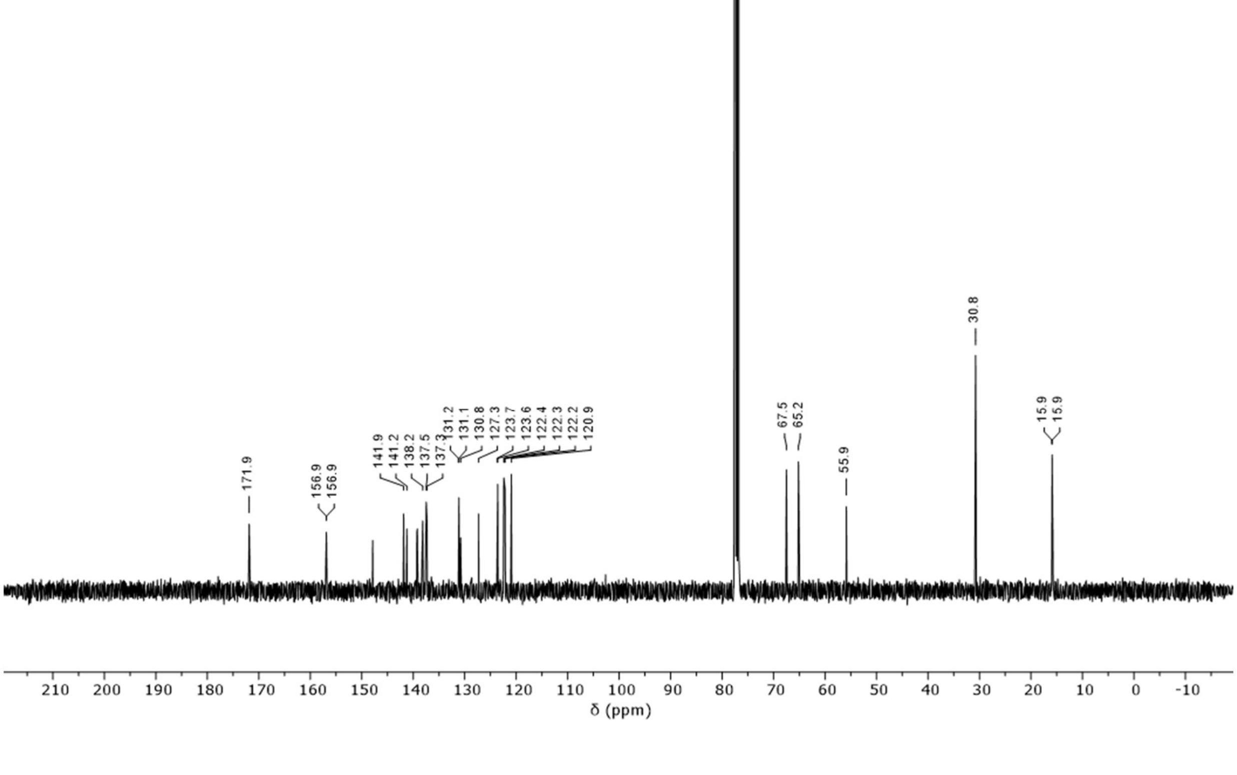

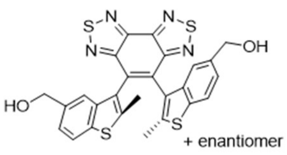

(±)-**7C**$_2$

^{1}H (400 MHz, $CDCl_3$)

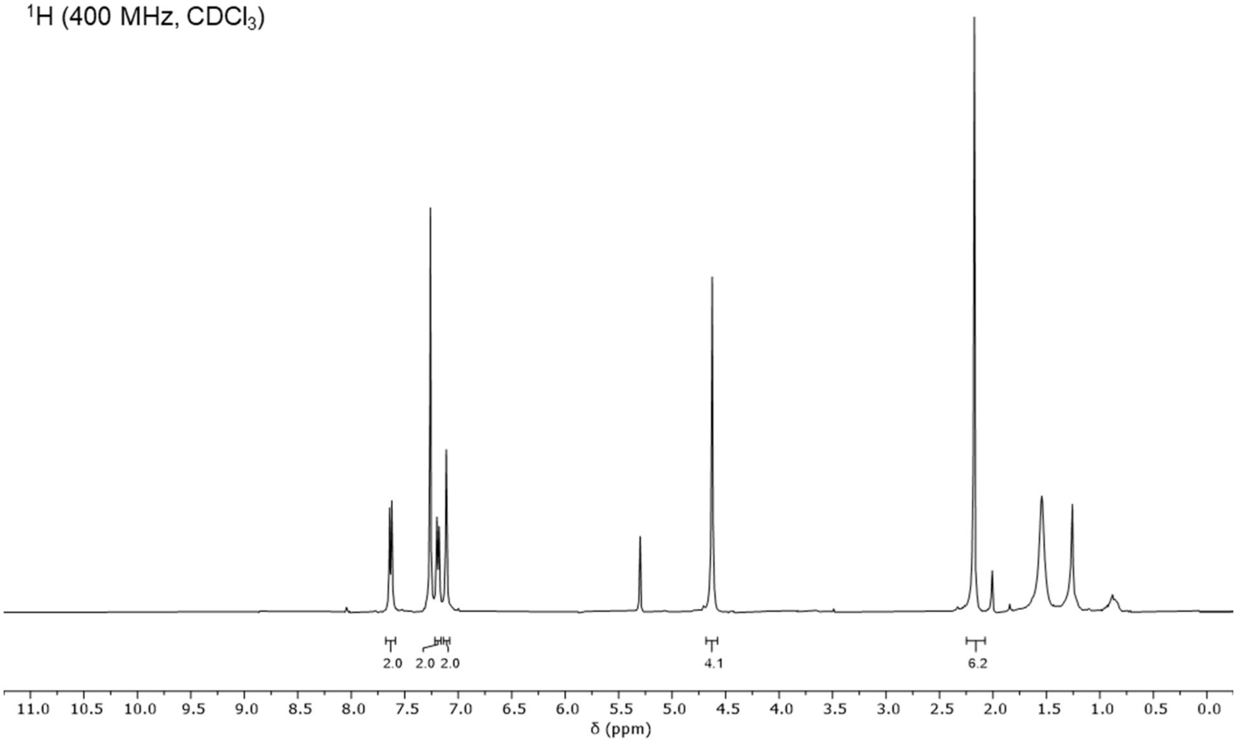

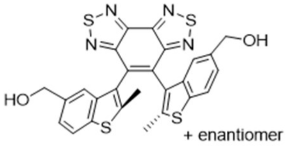

(±)-**7C**$_2$

^{13}C (100 MHz, $CDCl_3$)

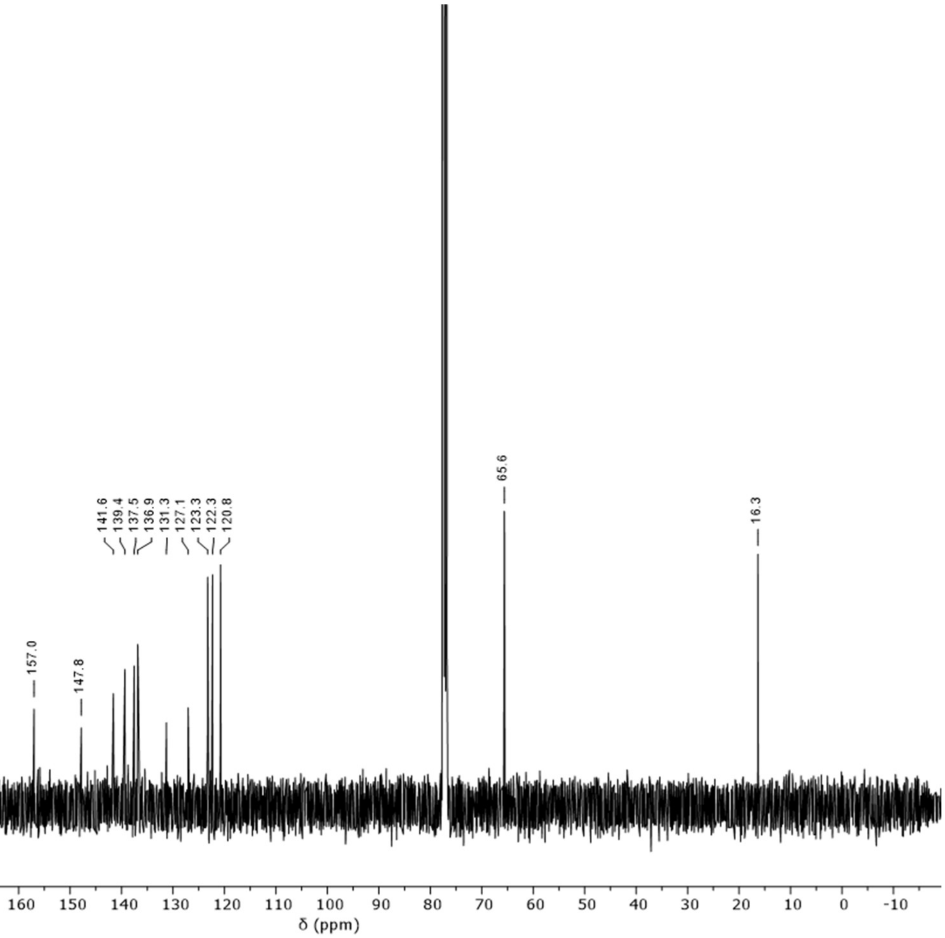

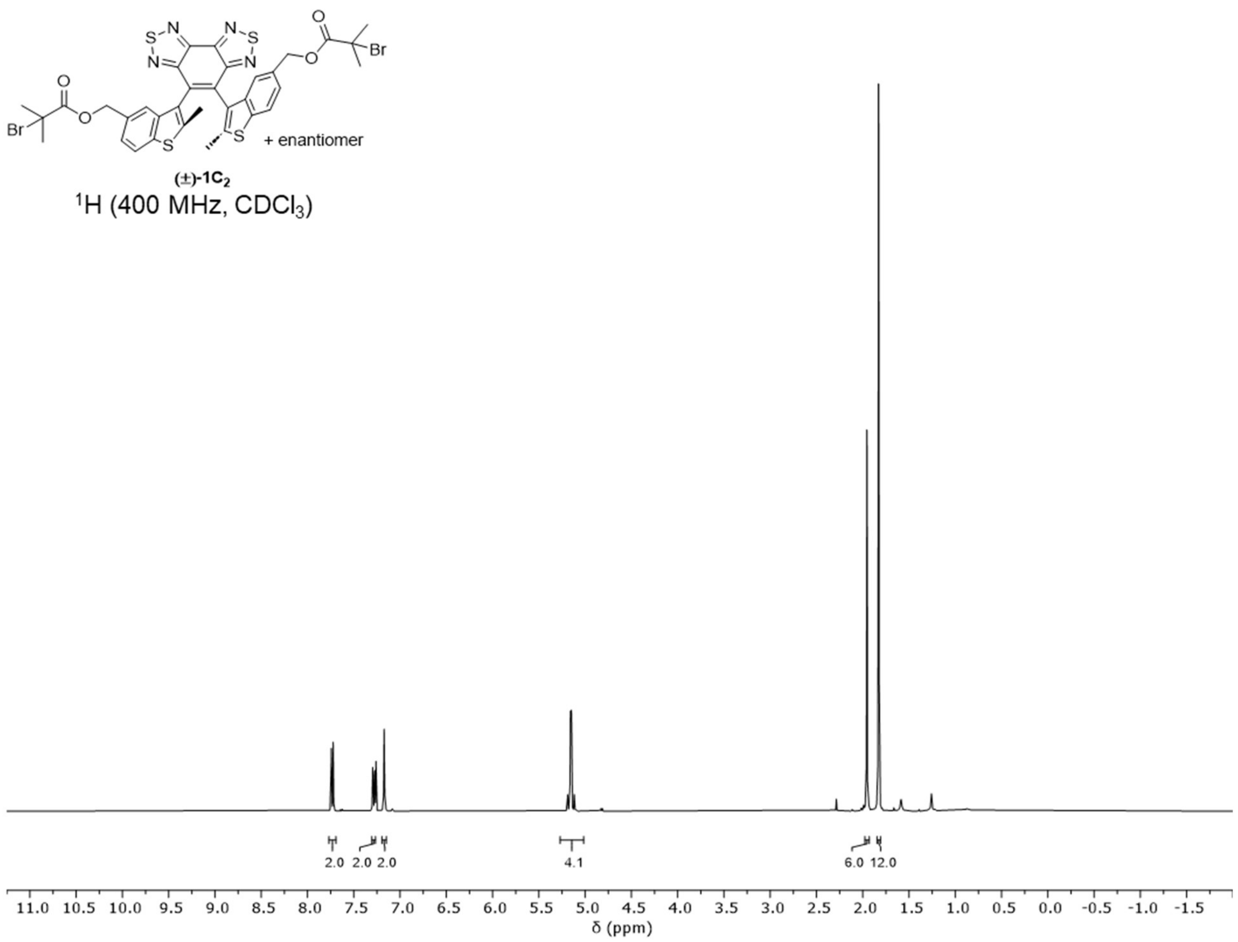
+ enantiomer
(±)-1C2
1H (400 MHz, CDCl3)
2.0 2.0 2.0
4.1
6.0 12.0
δ (ppm)

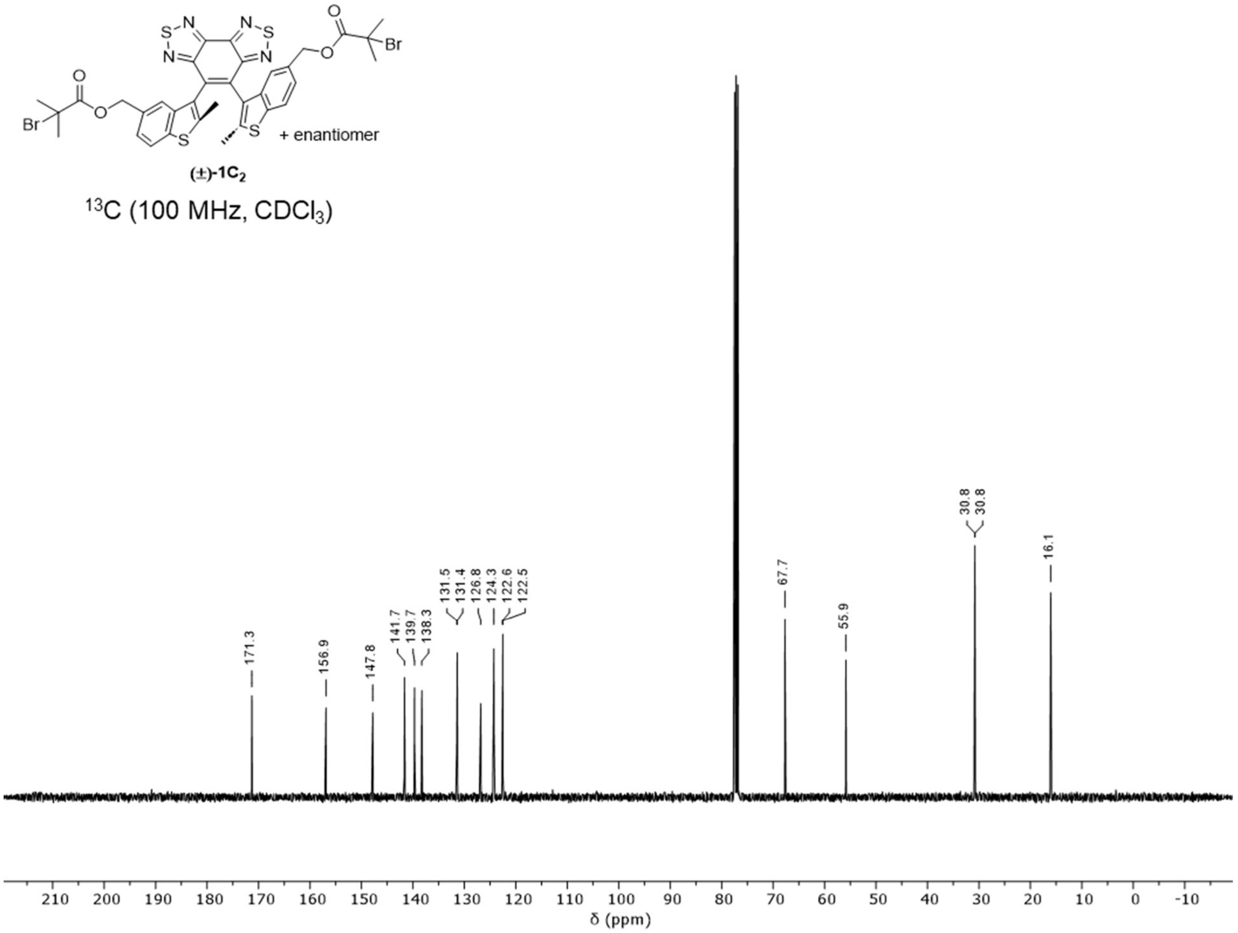
+ enantiomer
(±)-1C2
13C (100 MHz, CDCl3)
171.3
156.9
147.8
141.7
139.7
138.3
131.5
131.4
126.8
124.3
122.6
122.5
67.7
55.9
30.8
30.8
16.1
δ (ppm)

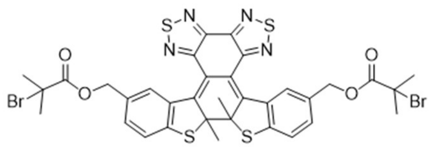

(±)-$1C_2^{closed}$

^{1}H (400 MHz, $CDCl_3$)

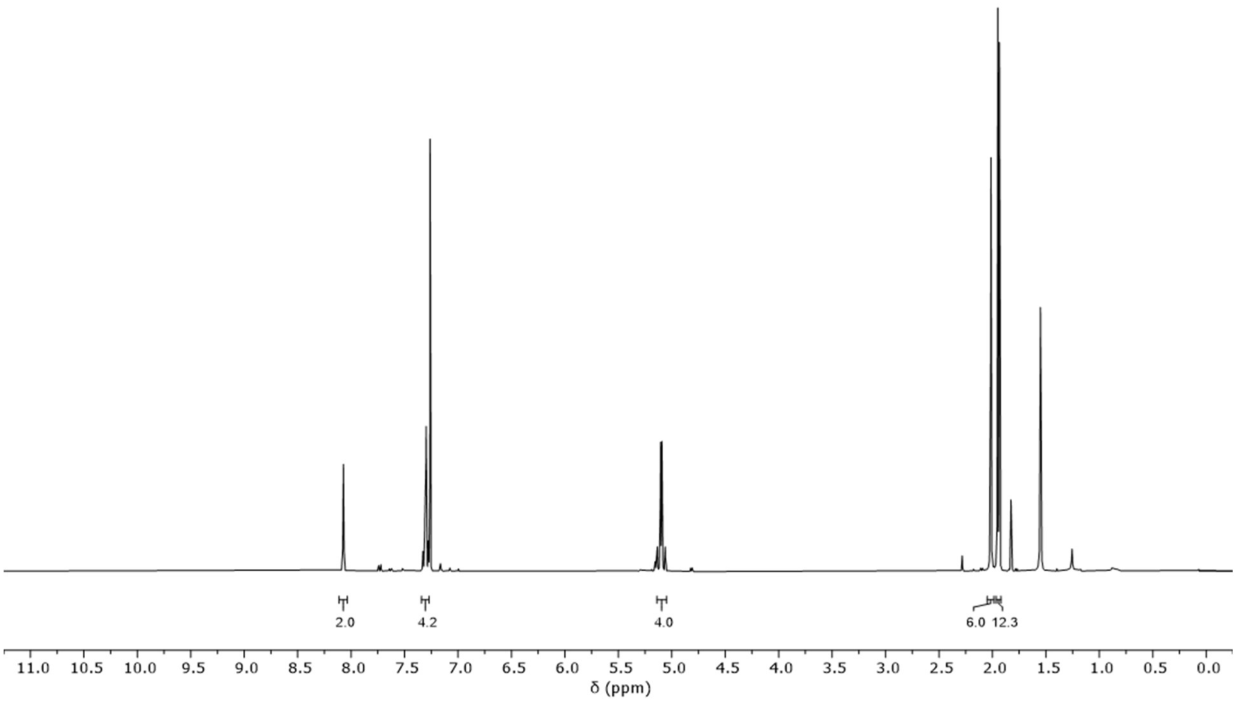

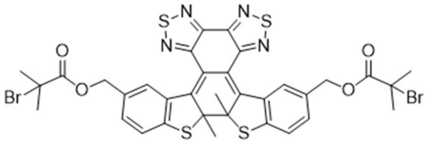

(±)-$1C_2^{closed}$

^{13}C (100 MHz, $CDCl_3$)

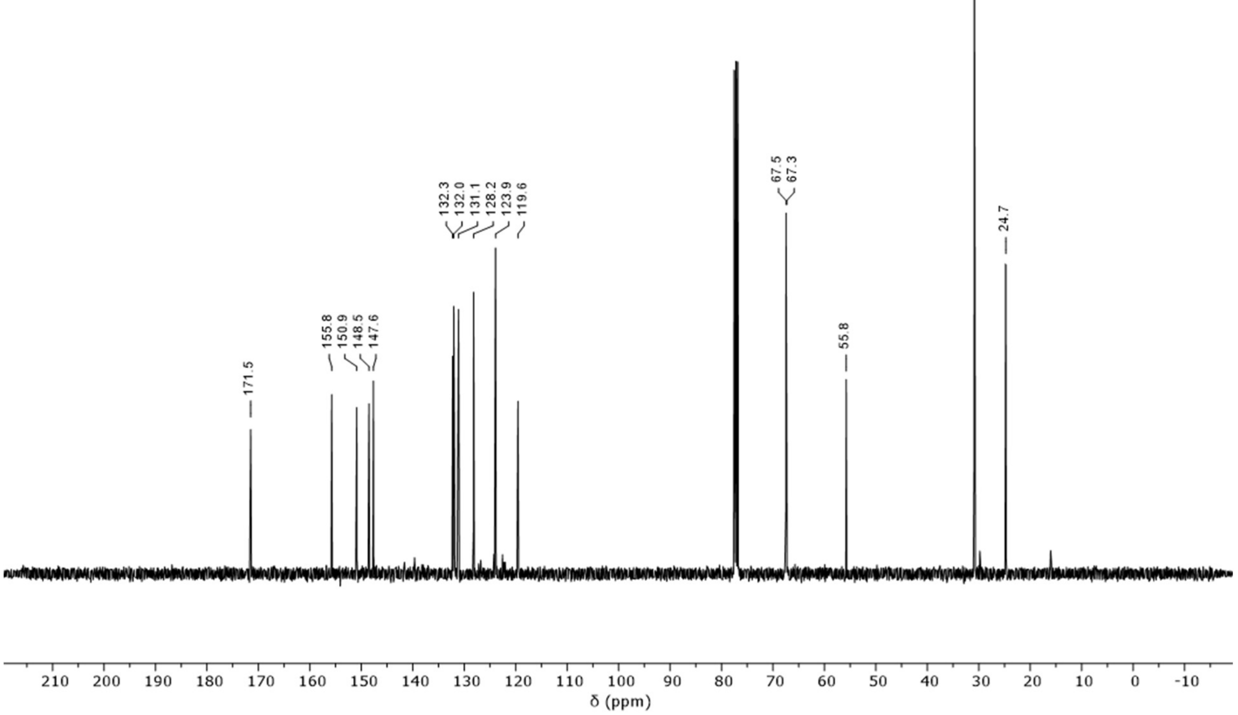

7. Single Crystal X-ray Diffraction

Crystals of **7σ** were grown by slow diffusion of hexanes into a solution of **7σ** in a near 1:1 mixture of chloroform and ethanol. Low-temperature X-ray diffraction data for **7σ** were collected on a Rigaku XtaLAB Synergy diffractometer coupled to a Rigaku Hypix detector with Cu Kα radiation (λ = 1.54184 Å), from a PhotonJet micro-focus X-ray source at 104 K. The diffraction images were processed and scaled using the CrysAlisPro software (CrysAlisPro; Rigaku OD, The Woodlands, TX, **2015**). The structures were solved through intrinsic phasing using SHELXT (Sheldrick, G. M. *Acta Cryst*. **2015**, A71, 3-8.) and refined against F^2 on all data by full-matrix least squares with SHELXL (Sheldrick, G.M. *Acta Cryst*., **2008**, A64, 112-122.) following established refinement strategies (Müller, P. Crystallography Reviews 2009, 15, 57-83.). All non-hydrogen atoms were refined anisotropically. All hydrogen atoms bound to carbon were included in the model at geometrically calculated positions and refined using a riding model. The isotropic displacement parameters of all hydrogen atoms were fixed to 1.2 times the Ueq value of the atoms they are linked to (1.5 times for methyl groups). Special refinement details: **7σ** crystallizes in the triclinic space group P-1 (#2) with one molecule per asymmetric unit; the asymmetric unit also contains 0.5 disordered solvent EtOH molecule that was included in the unit cell but could not be satisfactorily modeled. Therefore, it was treated as diffuse contributions to the overall scattering without specific atom positions using the solvent mask routine in Olex2 (Dolomanov, O.V.; Bourhis, L.J.; Gildea, R.J.; Howard, J.A.K.; Puschmann, H. *J. Appl. Cryst.*, **2009**, 42, 339-341) The -OH moieties of the molecule are disordered and were modeled with either two- or three-components. Because of the heavy disorder, the H-atoms couldn't be found in the difference map and were included in the model at geometrically calculated positions.

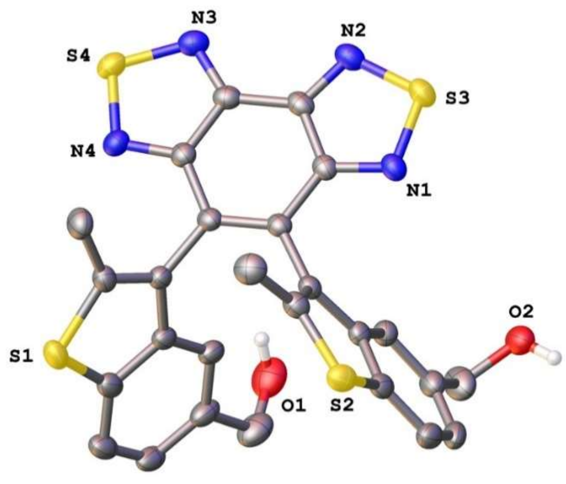

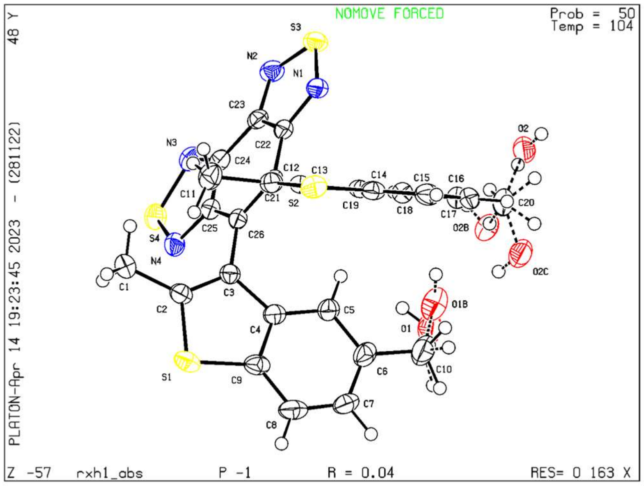

Displacement ellipsoid plot of **7σ** (50% probability level). H atoms not bound to oxygen and structural disorder have been omitted for clarity.

Crystal data and structure refinement for Rxh1 (7σ)

Identification code	rxh1_abs	
Empirical formula	C27 H21 N4 O2.50 S4	
Formula weight	569.72	
Temperature	104(3) K	
Wavelength	1.54184 Å	
Crystal system	Triclinic	
Space group	P-1	
Unit cell dimensions	a = 8.65390(10) Å	α = 102.1120(10)°.
	b = 10.50530(10) Å	β = 98.5100(10)°.
	c = 14.7973(2) Å	γ = 102.9170(10)°.
Volume	1254.93(3) $Å^3$	
Z	2	
Density (calculated)	1.508 Mg/m^3	
Absorption coefficient	3.789 mm^{-1}	
F(000)	590	
Crystal size	0.125 x 0.112 x 0.052 mm^3	
Theta range for data collection	3.120 to 80.100°.	
Index ranges	-11<=h<=11, -13<=k<=13, -18<=l<=18	
Reflections collected	52810	
Independent reflections	5411 [R(int) = 0.0481]	
Completeness to theta = 67.684°	99.9 %	
Absorption correction	Gaussian	
Max. and min. transmission	1.000 and 0.696	
Refinement method	Full-matrix least-squares on F^2	
Data / restraints / parameters	5411 / 11 / 363	
Goodness-of-fit on F^2	1.093	
Final R indices [I>2sigma(I)]	R1 = 0.0404, wR2 = 0.1084	
R indices (all data)	R1 = 0.0423, wR2 = 0.1100	
Extinction coefficient	n/a	
Largest diff. peak and hole	0.410 and -0.461 e.$Å^{-3}$	
